# Perspectives on the Limits and Clinical Alignment of Medical AI from Population Statistics to Individual Care

**DOI:** 10.3390/bioengineering13091034

**Published:** 2026-09-05

**Authors:** Milan Toma, David Yusupov

**Affiliations:** Laboratory of Algorithmic Medicine, Department of Osteopathic Manipulative Medicine, College of Osteopathic Medicine, New York Institute of Technology, Old Westbury, NY 11568, USA; dyusup02@nyit.edu

**Keywords:** clinical artificial intelligence, diagnostic classification, predictive prognostics, combinatorial analysis, cognitive surrender, population statistics, human-machine collaboration, clinical judgment

## Abstract

The clinical integration of artificial intelligence has outpaced the development of robust evaluative frameworks, raising critical safety concerns. This perspective establishes a clear taxonomy distinguishing probabilistic language models from deterministic classifiers and applies a multi-dimensional combinatorial model to calculate the requirements for complete diagnostic coverage. Our analysis demonstrates that comprehensive diagnostic coverage requires between 50,000 and 150,000 distinct, task-specific classifiers under subspecialty-level clinical granularity; conservative aggregated estimates (4500–18,750 binary classifiers) do not reflect the multiplicative expansion introduced by subtype differentiation, severity staging, temporal variants, demographic stratification, and equipment variation, whereas currently cleared devices cover less than one percent of this clinical space. More fundamentally, although population-trained models can generate conditional patient-specific risk estimates when predictors are informative and calibration is adequate, these statistical parameters optimized on population-scale data cannot provide the categorical certainty required for individual diagnostic decisions, which is a gap that clinical judgment must bridge. Because clinical AI tools are inherently statistical and perform reliably only on common, highly represented presentations while failing on rare, atypical cases rare in their training data, attempting to automate routine tasks leaves human clinicians with only the most challenging diagnostics. Furthermore, selective automation of these low-complexity cases introduces severe occupational hazards, including cognitive surrender, diagnostic complacency, and rapid expertise atrophy. Rather than pursuing the computationally and logistically unfeasible goal of complete diagnostic classification, developers should prioritize predictive, prognostic trajectory modeling. This paradigm shift aligns the probabilistic nature of machine learning with clinical utility, reinforcing clinical judgment as the irreplaceable diagnostic integrator.

## 1. Introduction

These perspectives examine two fundamental categories of artificial intelligence (AI) systems deployed in clinical medicine: “probabilistic language models” (e.g., large language models, LLMs) and “deterministic classification algorithms” (i.e., systems producing reproducible outputs for identical inputs given fixed model parameters; e.g., traditional machine learning, ML) [1]. The analysis of operational mechanisms, shared limitations, and appropriate integration into clinical workflows draws on established frameworks emphasizing that healthcare machine learning must shift from model development toward deployment, monitoring, and data-centric evaluation [2]. Responsible translation from algorithm development to patient care requires attention to safety, fairness, and robustness [3]. The framework presented here aligns with published quality criteria for AI-based prediction models spanning development, evaluation, and implementation [4].

Critically, this study presents a combinatorial analysis demonstrating the practical impossibility of achieving detailed diagnostic coverage through task-specific classifiers, and argues that the true clinical value of these systems lies in ’predictive’ rather than ‘classificatory’ applications.

### 1.1. Definitions, Prior Taxonomies, and Categorical Framework

Every presentation about artificial intelligence in medicine (i.e., every conference talk, every workshop, every poster session, every journal article) should begin by answering a simple question: ‘What kind of AI are we discussing?’ This clarification has become necessary because the term “AI” has been rendered nearly meaningless through imprecise usage. For much of the general public, and indeed for many clinicians, “AI” has become synonymous with chatbots, i.e., conversational systems like ChatGPT, Claude, or Gemini that generate human-like text responses. This conflation is both historically amnestic and practically dangerous. It erases over half a century of AI research, from expert systems and neural networks to computer vision and signal processing, and dismisses the hundreds of task-specific diagnostic algorithms that have received regulatory clearance for clinical use. The chatbot that drafts a clinic note operates on fundamentally different principles than the algorithm that detects diabetic retinopathy, measures cardiac ejection fraction, or identifies malignant skin lesions. Conflating these technologies obscures their different capabilities, different failure modes, and different appropriate use cases.

To appreciate the boundaries of this categorical distinction, it is necessary to position our approach against existing taxonomies and perspective papers on medical AI limitations, such as those establishing initial classification criteria for clinical algorithms [2,3,4] or framing general translation barriers [5]. While these prior frameworks offer invaluable categorization of model types, data requirements, and broad ethical principles, they typically treat the diagnostic challenge as a flat, scale-free problem. Our framework departs from these taxonomies in two fundamental ways: first, we explicitly analyze the population-to-individual epistemological gap that prevents statistical expectations from guaranteeing categorical bedside decisions; and second, we introduce a multi-dimensional combinatorial model that calculates the exact multiplicative expansion of subspecialty-level clinical requirements. By moving beyond descriptive categorization, this perspective provides a quantitative mechanism to evaluate the feasibility of comprehensive diagnostic automation.

#### 1.1.1. Probabilistic Models (Large Language Models)

Probabilistic AI systems, exemplified by large language models (LLMs), operate through statistical pattern recognition across vast corpora of text data [6]. These systems can encode clinical knowledge and answer clinical-style questions, though evaluation still requires human review for factuality, comprehension, reasoning, possible harm, and bias [7]. The language model operates by calculating the conditional probability of each subsequent word in a sequence based on the context of the preceding words. This probability distribution is mapped across the system’s entire vocabulary using parameters that are learned and optimized through training on massive, population-scale textual datasets.

In clinical contexts, LLMs function as sophisticated pattern-matching engines that mirror but do not replicate initial clinical reasoning [8]. When queried with symptom presentations, these systems retrieve and synthesize patterns from training data representing aggregate clinical experiences, producing outputs that statistically approximate differential diagnoses without understanding underlying pathophysiology [9]. LLMs may generate fluent, statistically plausible text while raising risks from scale, data limitations, and ungrounded language generation [8]. As Krakauer et al. explain, rather than being truly smart, the AI is just running a massive, brute-force guessing game using billions of data points, meaning it has “skills” but not “smarts” [10]. In contrast, they argue that real intelligence means finding simple, elegant shortcuts, like using basic analogies and high-level ideas to solve a wide variety of new problems quickly [10].

As summarized in Table 1, these systems require physician oversight due to hallucination risk, safety concerns, and the fundamental distinction between pattern recognition and verified clinical judgment [9,11]. Recent studies of LLMs applied to differential diagnosis using challenging clinical cases demonstrate that differential diagnosis is closer to real clinical reasoning than simple multiple-choice benchmarking, positioning LLMs as hypothesis generators and clinician aids rather than final diagnostic arbiters [12]. Conversational diagnostic AI evaluations that include history-taking and diagnostic dialogue highlight the need to test AI in workflow-like settings rather than static question-answering formats [13]. Studies of generalist medical language models suggest that large medical models may support diagnostic decision-making while still requiring clinician interpretation and validation [14]. Ethical and regulatory reviews of LLMs in medicine emphasize safety, accountability, bias, privacy, and governance considerations [15].

Randomized clinical trial evidence evaluating LLM assistance for diagnostic reasoning has found that access to an LLM did not significantly improve physician diagnostic reasoning in certain tested settings [16]. Evaluation of multiple off-the-shelf LLMs on clinical reasoning tasks has shown stronger performance on some final diagnosis or management tasks but weaker performance on differential-diagnosis reasoning with incomplete information [17]. Cross-sectional studies testing whether LLM medical benchmark performance reflects logical medical reasoning or pattern recognition support the claim that LLM outputs can appear correct while still requiring clinician verification and critical appraisal [18]. The rapid growth of peer-reviewed clinical LLM studies from 2022 onward has created an increasingly difficult evidence base to appraise, justifying the urgent need for critical appraisal standards [19]. Single-case evaluation of leading multimodal LLMs for radiological image interpretation has demonstrated that these systems are susceptible to severe, fundamental diagnostic failures and high evaluative variance. Even when models reached a consensus, clinically meaningful variability persisted in acuity characterization and anatomical localization, while cross-evaluation exposed significant disagreements in radiological ground truth, self-evaluation biases, and highly inconsistent grading stringency among models [20]. These findings demonstrate that current multimodal LLMs exhibit unacceptable diagnostic variability and evaluative inconsistency for autonomous clinical deployment, and that healthcare applications requiring reliable image interpretation should prioritize validated, task-specific machine learning systems over general-purpose language models [20].

#### 1.1.2. Deterministic Classification Models (Machine/Deep Learning)

Deterministic classification systems employ supervised learning algorithms to map patient features to discrete diagnostic categories. In straightforward applications like logistic regression, this boundary is established by evaluating a weighted combination of input variables to generate a probability score. In deep neural networks, this process is scaled through multiple layers of nonlinear computations. The underlying model parameters are optimized iteratively using gradient descent on population-level training data to minimize classification errors.

These systems are typically trained for specific diagnostic tasks such as detecting pneumonia on chest radiographs [21], detecting radiograph fractures [22], identifying diabetic retinopathy in fundoscopic images [23], classifying arrhythmias from electrocardiogram waveforms [24], to name a few. Systematic reviews and meta-analyses comparing deep learning with healthcare professionals in medical imaging have reported promising pooled accuracy but emphasized limited high-quality external validation and comparable human–AI testing [25]. Systematic reviews of studies comparing AI with clinicians have found major limitations in study design, reporting, and risk of bias, supporting the argument that diagnostic AI evidence must be judged by clinical trial quality rather than headline accuracy [26]. Further systematic review and meta-analysis of diagnostic accuracy of deep learning in medical imaging highlights that poor design and reporting can overestimate algorithm performance [27].

Classification algorithms are particularly susceptible to methodological errors that artificially inflate reported accuracy; studies examining data leakage and feature selection effects have demonstrated that improper data partitioning or feature engineering can produce performance metrics that fail to generalize, thereby compromising clinical applicability [28]. Shortcuts to high accuracy carry unacceptable risks: spurious correlations and dataset biases can produce models that perform well on validation datasets yet fail catastrophically in clinical practice [29]. To prevent these failures, researchers advocate for reclaiming interpretable machine learning architectures over post-hoc explainability, establishing a set of fundamental design principles to bridge rule-based logic and deep networks safely [30,31].

While termed “deterministic” due to their reproducible outputs for identical inputs given fixed model parameters, these systems, like their probabilistic LLM counterparts, are fundamentally statistical tools (see Table 2): the learned parameters encode ‘population-wide statistical associations’ rather than mechanistic understanding of disease processes. This shared epistemological foundation is examined in Section 3.

Transparent reporting of deterministic classifier studies should follow established guidelines such as STARD-AI, which addresses reporting of datasets, index tests, evaluation, bias, applicability, and generalizability [32], and the CLAIM checklist for radiology-specific reporting standards covering data, model development, validation, performance, and reproducibility [33].

These distinct architectural paradigms establish the fundamental divergence between probabilistic language generation and task-specific deterministic classification [1]. While large language models calculate conditional probability distributions over heterogeneous vocabulary sets to synthesize conversational text, deterministic classifiers learn mathematical decision boundaries on highly structured, expert-labeled datasets to output discrete categorical predictions [6,32]. In essence, the former functions as a flexible, text-based hypothesis generator mimicking initial clinical reasoning, whereas the latter operates as a narrow, validated diagnostic test with quantified sensitivity and specificity tailored to a specific pathology [9,33].

Bridging the gap between these baseline definitions and safe clinical implementation requires a detailed comparative evaluation of their operational limits [2,4]. Conflating general-purpose verbal fluency with localized, task-specific diagnostic accuracy is historically inaccurate and clinically dangerous, as it obscures their completely different capabilities, failure modes, and regulatory baselines [20]. To establish clear boundaries of clinical utility, the following section systematically contrasts general-purpose language models and task-specific machine learning models across seven critical operational dimensions, providing a validated decision framework to guide healthcare leadership and developers through safe, appropriate integration [20,34].

## 2. Distinguishing LLMs from Task-Specific ML Models

Given the distinct operational characteristics of probabilistic LLMs and deterministic classification systems described above, a critical question arises for clinical deployment: when is each type of AI system appropriate? Recent empirical evidence provides guidance.

Hong et al. [20] evaluated five leading multimodal LLMs (GPT-5, Gemini 3 Pro, Llama 4 Maverick, Grok 4, and Claude Opus 4.5 Extended) on a standardized non-contrast head CT interpretation task. Their findings illustrate the clinical severity of these errors: one model misidentified an ischemic stroke as an intracerebral hemorrhage with incorrect lateralization, which would be a mistake with potentially catastrophic clinical implications, as the management of ischemic stroke differs fundamentally from that of intracerebral hemorrhage.

Most significantly, a novel cross-evaluation protocol wherein each LLM graded all five responses revealed that LLMs cannot reliably agree on radiological ground truth: one model interpreted the image as showing acute infarction with mass effect, while another concluded it depicted chronic infarction with atrophy, i.e., diametrically opposed interpretations with opposite therapeutic implications [20]. This ground truth disagreement, combined with observed self-evaluation bias and inconsistent grading standards, undermines proposals for using LLMs as automated quality assurance tools or as substitutes for validated classifiers.

**Figure 1:**
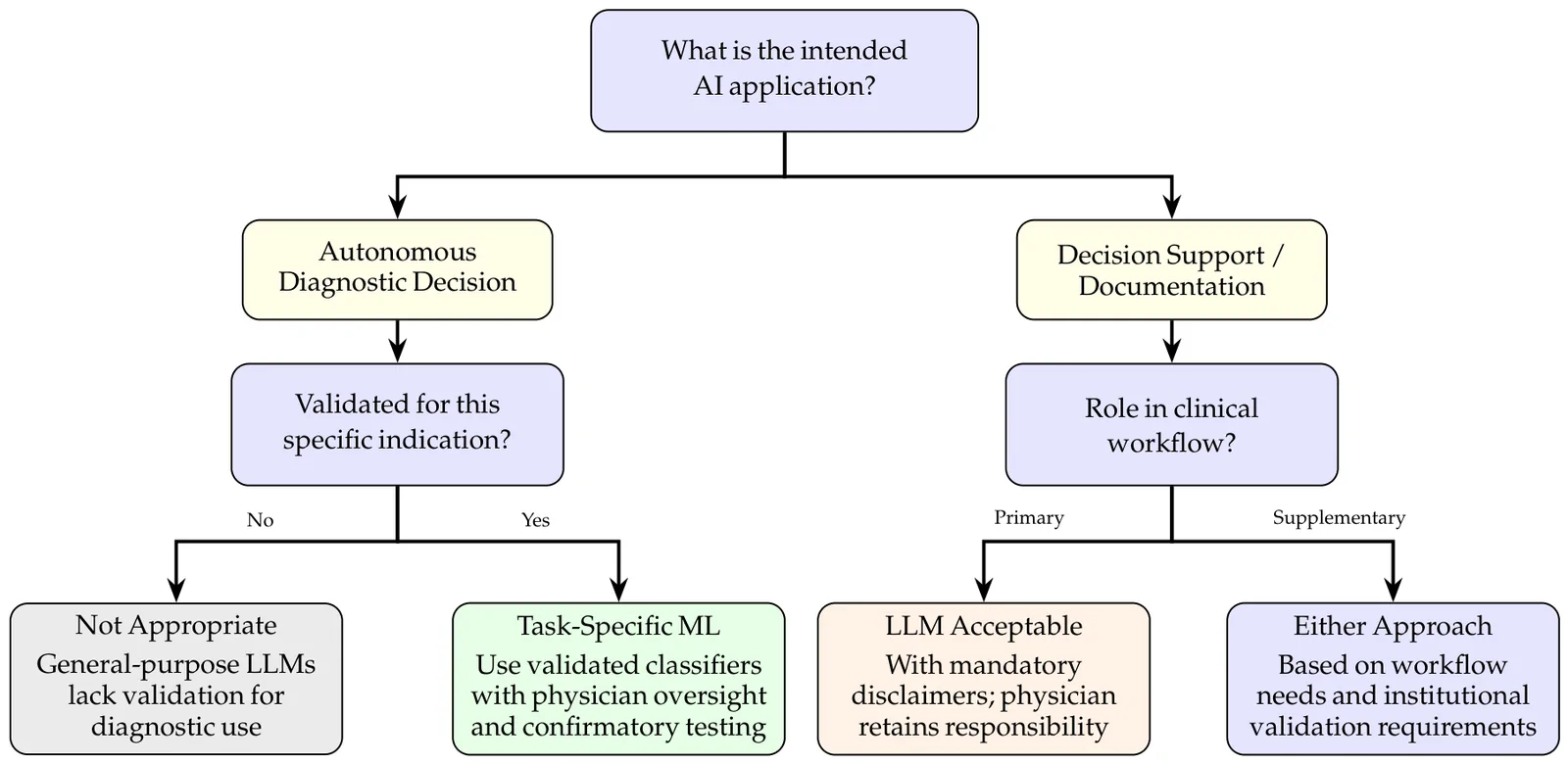
Decision framework for selecting between general-purpose LLMs and task-specific machine learning models in clinical settings. Autonomous diagnostic applications require validated task-specific models with physician oversight, while LLMs may serve supporting roles (documentation, literature synthesis) with appropriate disclaimers. This framework operationalizes the distinctions enumerated in Table 3.

Table 3 summarizes the key distinctions between general-purpose multimodal LLMs and task-specific machine learning models across seven critical dimensions relevant to clinical deployment. Additionally, Figure 1 provides a practical decision framework for determining when general-purpose LLMs versus task-specific ML models are appropriate in clinical settings, operationalizing the distinctions enumerated in Table 3.

## 3. The Shared Fundamental Limitation

Despite their architectural differences, both probabilistic and deterministic AI systems share an irreducible epistemological limitation: they are trained on population-wide data but deployed for individual patient care. External validation studies of deep learning pneumonia detectors across hospital systems have demonstrated variable generalization and evidence that models can learn hospital-specific confounders rather than true disease signal [38]. This creates a fundamental mismatch between the statistical nature of AI outputs and the individualized nature of clinical decision-making.

### 3.1. The Statistical Foundation of All AI Systems

Every artificial intelligence system configures its internal parameters by optimizing a mathematical function over a specific training dataset. This process minimizes the average prediction error on the training examples while incorporating a secondary penalty term to prevent the model from overfitting to the historical data, ensuring the model remains applicable to the broader population distribution.

The learned parameters encode statistical regularities present in the training database. When the model evaluates a new patient, its output represents a statistical expectation or average calculated across the training population distribution. This output does not constitute an individualized diagnosis tailored specifically to the unique patient sitting in front of the clinician.

For example, shortcut-learning studies have shown that AI models for radiographic COVID-19 detection can rely on confounding artifacts rather than disease signal and fail when moved to new hospitals [39]. Systematic review of machine learning models for COVID-19 diagnosis and prognosis using chest imaging found widespread methodological flaws and bias, concluding that models were not clinically useful at that time [40]. Systematic review of external validation in radiology deep learning found that most externally validated algorithms had at least some performance decrease, with many showing clinically meaningful drops [41]. The fundamental question is “Does this specific patient conform to the statistical patterns learned from the training population?” To address this challenge, auxiliary algorithms can be deployed alongside the primary model to evaluate patient inputs and flag instances where a specific individual’s clinical feature profile deviates substantially from the characteristics most represented in the training population. These secondary screening tools serve as algorithmic guardrails, alerting clinicians when a patient possesses atypical clinical presentations, unusual demographics, or rare combinations of variables that make the primary diagnostic classification unreliable.

### 3.2. Why Population Statistics Cannot Provide Categorical Certainty for Individual Outcomes

Consider a deterministic classifier trained to detect pneumonia on chest radiographs with 95% sensitivity and 90% specificity. These metrics describe aggregate, population-level performance. We acknowledge that such models can produce patient-specific conditional probability estimates (e.g., “given these imaging features, the posterior probability of pneumonia is 0.87”); the critical limitation is not that population statistics provide no individual-level information, but that they cannot warrant the categorical diagnostic certainty that clinical decision-making often demands. For instance, a clinical model with ninety-five percent sensitivity will correctly identify ninety-five percent of patients who actually have the disease, while a specificity of ninety percent means the model will correctly classify ninety percent of patients who do not have the condition.

When this classifier produces a positive result for a specific patient, the clinician faces an irreducible uncertainty: Is this patient among the 95% of true positives correctly identified, or among the 10% of true negatives incorrectly flagged? The population-level performance metrics provide no answer for the individual case.

More fundamentally, the classifier’s learned decision boundary reflects statistical patterns in the training population. Studies have demonstrated underdiagnosis bias in chest radiograph AI across underserved populations, supporting the claim that population-trained AI can systematically fail subgroups [42]. Shortcut-learning and confounding studies in hip-fracture prediction have shown that deep learning models can exploit patient and healthcare-process variables rather than fracture-specific signal [43]. Fairness and generalization studies in medical imaging AI have examined demographic encodings, shortcuts, and fairness discrepancies across internal and external test settings, providing evidence that fairness on one dataset may not generalize to real-world deployment [44].

Patients with atypical presentations, unusual anatomy, rare comorbidities, or demographic characteristics underrepresented in training data may fall in regions of feature space where the learned decision boundary poorly approximates the true underlying relationship. This is clearly demonstrated in skeletal imaging, where developmental anatomical differences like pediatric growth plates represent distinct imaging domains; classifiers trained on mixed-age populations can fail completely when cross-tested on pediatric wrist radiographs, resulting in a catastrophic collapse of diagnostic specificity [22].

### 3.3. Uncertainty Quantification and Boundary Alerts

To mitigate the risk of deploying population-level models to patients whose clinical features deviate from the training cohort, clinical decision support systems would benefit from the programmatic integration of real-time safeguards. Specifically, the development of robust uncertainty quantification methods can provide clinicians with an explicit measure of prediction reliability for each unique case, establishing a structured path toward enhanced and verified image classification performance [45]. Furthermore, incorporating automated flagging mechanisms that alert users when a patient’s input values fall outside the multidimensional feature ranges observed during training represents a critical safety threshold. These input boundary alerts can serve as an immediate cognitive speedbump, prompting clinical users to exercise heightened skepticism and perform independent verification before relying on algorithmic outputs.

### 3.4. The Irreducibility of Clinical Judgment

**Figure 2:**
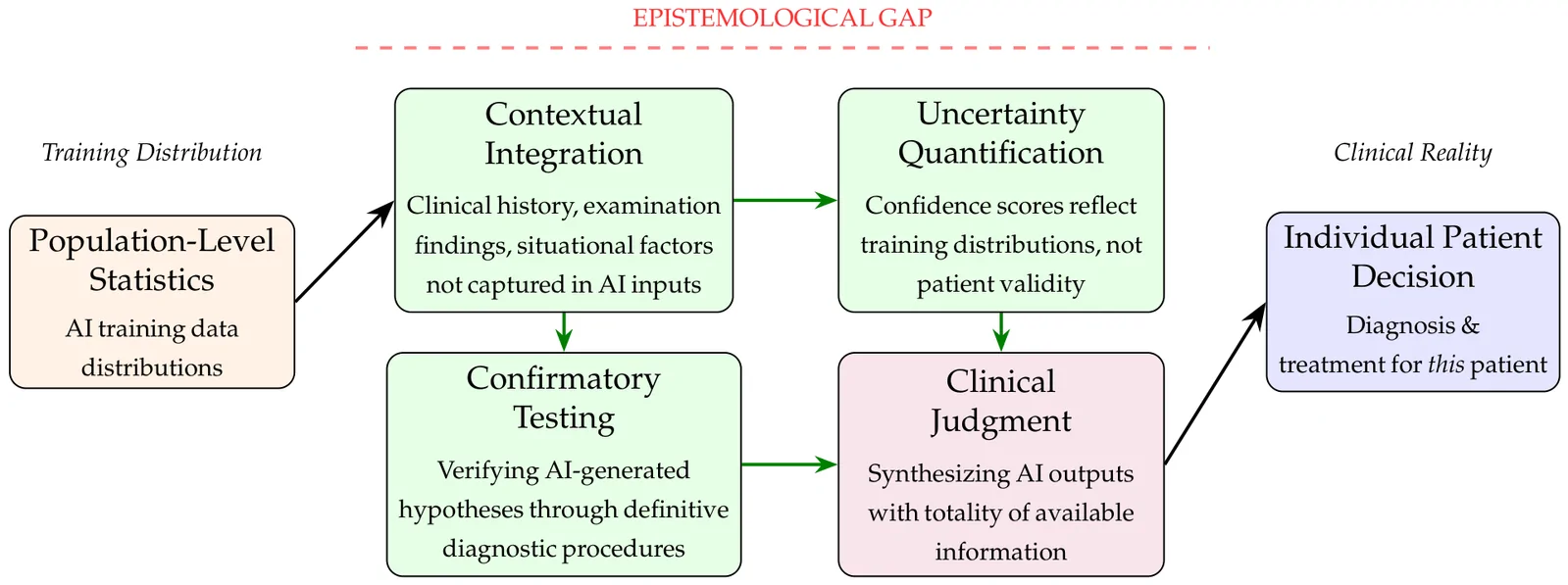
The irreducible gap between population-level AI outputs and individual patient decisions. Four physician-mediated processes (namely, contextual integration, uncertainty quantification, confirmatory testing, and clinical judgment) are required to bridge this epistemological divide. This gap is not a technical limitation to overcome but an inherent feature of applying statistical inference to individual cases. AI provides population-level pattern recognition; the physician translates this into patient-specific care through processes that cannot be automated.

This limitation is not a technical shortcoming to overcome through larger datasets or improved algorithms. It is an ‘epistemological reality’ inherent to statistical inference. The transition from population-level statistics to individual patient decisions requires bridging an irreducible gap through four essential physician-mediated processes, illustrated in Figure 2.

Calibration is critical for clinical prediction models; poor calibration can mislead decision-making, underscoring the importance of ensuring that risk estimates match observed outcomes in the target population [46]. Reviews of uncertainty quantification and abstention methods for healthcare machine learning directly support the point that AI confidence scores may not prove applicability to a specific patient and that safe systems should communicate uncertainty [47].

## 4. The Unchanged Diagnostic Workflow: AI as Informant, Not Arbiter

The preceding section established that both probabilistic LLMs and deterministic task-specific classifiers share a fundamental limitation: they are trained on population-level data but deployed for individual patient care (Section 2). This population-to-individual gap represents a constraint that no technical advancement can fully overcome. However, understanding ‘why’ physician judgment remains irreplaceable requires examining not merely the statistical properties of AI systems, but the cognitive architecture of clinical diagnosis itself, and the remarkable breadth of information that human physicians process, often without conscious awareness.

### 4.1. The Fundamental Diagnostic Process

**Figure 3:**
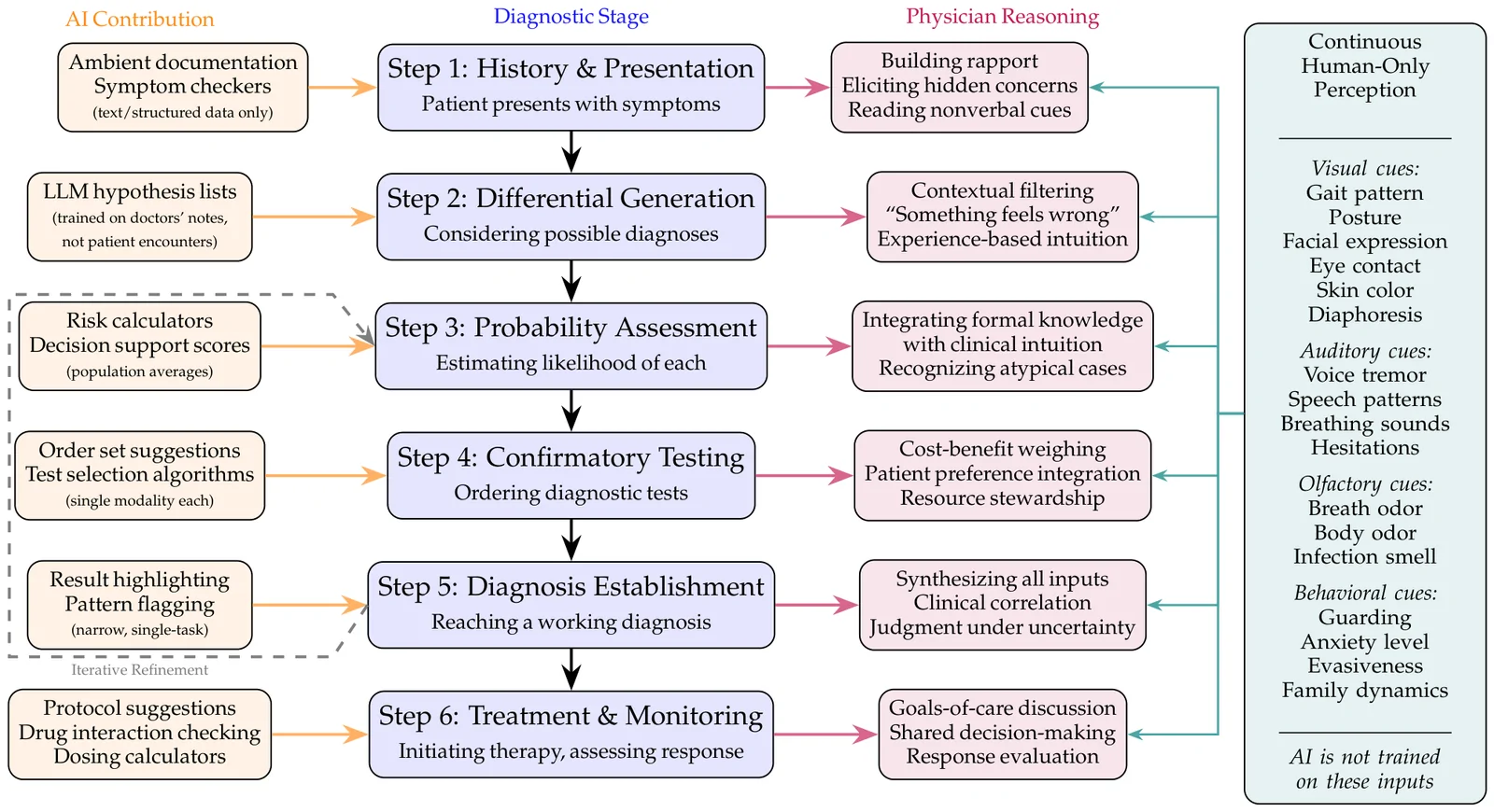
The fundamental diagnostic workflow in clinical medicine. Six stages progress from initial presentation through treatment, with iterative refinement between probability assessment and diagnosis establishment. Left column (orange) indicates AI system contributions, each narrowly trained on specific anatomical regions, data modalities, or pathologies. Center column (blue) shows the diagnostic stages. Right column (purple) identifies physician cognitive functions. Far right sidebar (teal) illustrates the continuous stream of human-only perceptual inputs (i.e., visual, auditory, olfactory, and behavioral cues) that inform clinical judgment throughout the encounter but lie entirely outside AI training data. This contrast demonstrates why diagnosis requires human integration of information streams that population-trained, narrowly-focused AI systems cannot access.

The core diagnostic workflow in clinical medicine follows a well-established structure that predates computational tools and will persist regardless of AI sophistication. This workflow (illustrated in Figure 3) is not merely a sequence of tasks that AI might eventually automate; it reflects the reality that diagnosis is an ‘inferential process’ requiring iterative evidence gathering, hypothesis testing, and integration of information streams that extend far beyond what any AI system can access.

Consider what happens when a physician first encounters a patient. Before a single word is spoken, the physician is already gathering diagnostic information: How does the patient walk into the room? Are their movements fluid or guarded? What is their posture: slumped, rigid, or relaxed? Are they making eye contact or avoiding it? Is their breathing labored, rapid, or calm? Do they appear diaphoretic? Is there a distinctive odor, e.g., the fruity breath of diabetic ketoacidosis, the fetor hepaticus of liver failure, the smell of alcohol or infection? Is the patient’s voice steady or trembling? Do they seem forthcoming or evasive? Are they accompanied by family members whose expressions convey concern, frustration, or fear?

This stands in stark contrast to how AI systems operate. Each AI tool is trained on a narrow slice of medical reality: a chest X-ray classifier sees only chest radiographs; a skin lesion detector sees only dermoscopic images; an ECG analyzer sees only electrical tracings; an LLM processes only the text of clinical notes. No AI system integrates the smell of a patient’s breath with the tremor in their voice, the way they guard their abdomen, and the worried glance exchanged between family members. AI systems are, by design, narrowly focused, i.e., trained on specific anatomical regions, specific imaging modalities, specific pathologies, or specific documentation patterns. The human physician, by contrast, is a generalist sensor array, continuously integrating conscious observations with subconscious pattern recognition developed over years of clinical experience.

Each stage of the diagnostic workflow involves cognitive operations that resist automation for reasons that are principled rather than merely technical. History-taking requires building trust and eliciting information that patients may not recognize as clinically relevant, or may be reluctant to share. Differential generation demands not just pattern-matching but contextual filtering based on patient-specific factors that may never appear in structured data. Probability assessment involves integrating formal knowledge with clinical intuition, e.g., the experienced physician’s sense that “something is wrong” even when objective findings are unremarkable. Confirmatory testing requires weighing costs, benefits, and patient preferences. Diagnosis establishment synthesizes test results with clinical context in ways that population statistics cannot prescribe. Treatment planning necessitates goals-of-care discussions and shared decision-making that are fundamentally interpersonal.

Figure 3 maps AI contributions alongside physician-essential functions at each stage, while also illustrating the continuous stream of human-only perceptual inputs that inform clinical judgment throughout the encounter. This analysis provides the conceptual foundation for the combinatorial analyses that follow: even if unlimited AI development resources were available, the transition from population-level statistics to individual patient decisions would still require physician judgment to bridge the irreducible gap between what is statistically likely and what is true for the specific patient.

### 4.2. Where AI Enters the Workflow

AI systems are being integrated at multiple points in this workflow, but the integration points determine appropriate use (Table 4):

### 4.3. The Principle of Physician-Machine Collaboration

Emerging evidence suggests that physician-machine collaboration outperforms either alone. Human–AI collaboration studies in skin cancer recognition have found that AI support can improve clinician performance, while poor AI support can mislead clinicians [48]. Human-centered deployment studies of diabetic-retinopathy AI in clinics have shown that workflow, environment, staff roles, and patient experience affect real-world AI performance, demonstrating that clinical AI is not just a model problem but a deployment problem [49]. Randomized clinical vignette studies have shown that standard AI can improve diagnostic accuracy but biased AI can worsen clinician performance, and explanations did not fully prevent harm [50]. The risk that clinicians may over-rely on AI-driven decision support, termed automation bias, strengthens the need for physician oversight, verification, and critical appraisal [51]. Sociotechnical reviews argue that clinical AI should complement physicians’ diagnostic reasoning and decision-making activities rather than focus only on benchmark performance [5]. The core diagnostic act, integrating AI outputs with clinical context, ordering confirmatory testing, and making final judgment, remains the physician’s domain. This is not a temporary limitation awaiting technological solution; it reflects the fundamental nature of individual patient care.

### 4.4. Complementary Error Profiles in Human-Machine Classification

**Figure 4:**
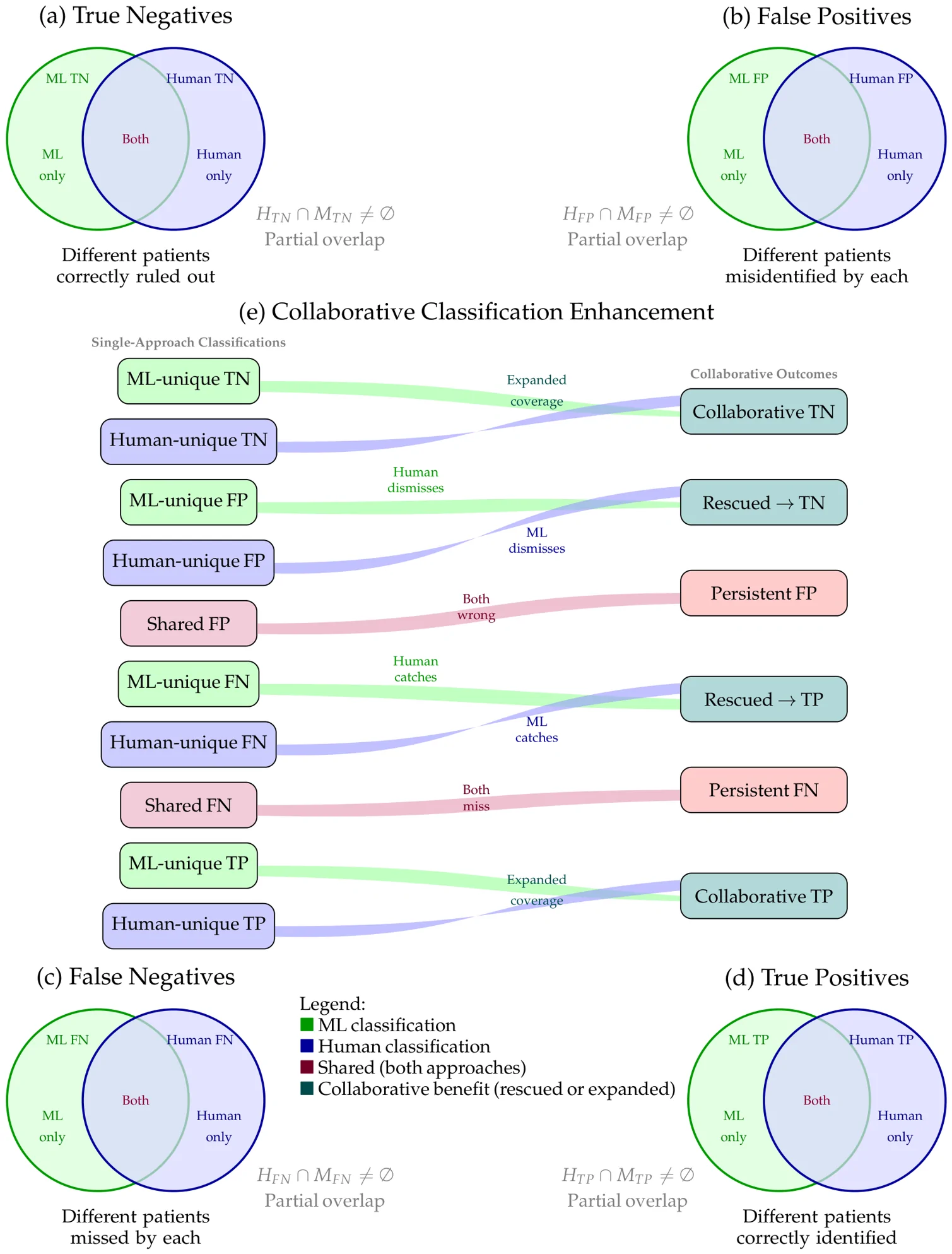
Complementary classification profiles and collaborative enhancement in human–machine classification, arranged in standard confusion matrix layout. Peripheral panels (**a**–**d**): Venn diagrams showing partial overlap in all four classification categories; human and machine approaches correctly classify, and misclassify, different patients. Central panel (**e**): Sankey diagram showing how collaboration benefits from partial overlap: correct classifications unique to one approach expand collaborative coverage, while errors unique to one approach are rescued by the other.

The principle that physician–machine collaboration outperforms either party alone can be understood through analysis of classification outcomes as demonstrated in Figure 4. When a classification algorithm renders a determination on clinical data, the result falls into one of four categories: true positive, true negative, false positive, or false negative. Human physicians making the same diagnostic determination produce outcomes in these identical categories. However, the populations of patients assigned to each category by machines versus humans exhibit systematically different overlap patterns, and understanding these patterns clarifies why collaborative workflows provide clinical value that neither approach achieves independently.

For purposes of this analysis, assume that a well-trained classification algorithm exhibits higher sensitivity and specificity than an unaided human reviewer for a given diagnostic task. This assumption may be justified in many clinical contexts, but it remains an assumption; the relationships described below follow from this premise rather than from any claim of universal algorithmic superiority. Crucially, higher aggregate sensitivity or specificity establishes only that one approach’s correct-classification set is larger, not that it fully contains the other’s correct-classification set. A machine achieving 95% sensitivity and a human achieving 85% sensitivity may nonetheless correctly identify partially different patient populations, i.e., the machine catches some cases the human misses, while the human catches some cases the machine misses. This partial overlap in correct classifications, rather than a subset relationship, characterizes the realistic relationship between human and machine performance (Figure 4a,d). The same partial-overlap structure applies to classification errors: human and machine false positives and false negatives represent overlapping but non-identical patient populations (Figure 4b,c).

The relationship changes fundamentally when examining classification errors. False positives, comprising patients who lack the pathological condition but are incorrectly flagged as positive, exhibit only partial overlap between human and machine classifications. The algorithm and the human reviewer make different mistakes on different patients. The classification algorithm may flag imaging artifacts, anatomical variants, or benign findings that experienced human reviewers would immediately dismiss. Gastroenterologists reviewing AI-flagged colonoscopy images frequently encounter this phenomenon, spending time examining regions that the algorithm identified as suspicious but that the experienced endoscopist recognizes as mucosal folds, stool residue, or reflection artifacts. A prospective image-based study comparing a computer-aided detection (CADe) system with experienced endoscopists found comparable false positive rates between the two approaches (specificity 86.64% vs. 85.41%, p=0.79), confirming that both humans and machines generate false positives in polyp detection, though the clinical workflow implicitly assumes these represent different error populations requiring human adjudication [52].

However, the human reviewer also generates false positives that differ from those of the algorithm. Confirmation bias, anchoring on initial impressions, or pattern completion based on incomplete data may lead the human to flag findings that the algorithm correctly classifies as negative. Notably, CADe false positive rates vary with imaging conditions: under optimal visualization (fully inflated colonic lumen), CADe systems achieve higher specificity than human endoscopists (95.69% vs. 86.70%, p=0.04), but under suboptimal conditions (deflated lumen), CADe specificity drops below human performance (77.59% vs. 84.11%, p=0.24) [52]. This context-dependent performance underscores that the partial overlap in false positive populations shifts depending on clinical conditions. The two circles representing human and machine false positives overlap only partially, with each containing errors unique to that classification approach.

False negatives exhibit the same partial-overlap pattern. Patients who have the pathological condition but are incorrectly classified as negative constitute different populations depending on whether classification is performed by human or machine. The algorithm may miss atypical presentations, cases with features poorly represented in training data, or findings that require integration of clinical context inaccessible to the image-only classifier. The human may miss subtle findings, findings in anatomical regions receiving less visual attention, or findings encountered late in a session when vigilance has decreased. Critically, these two sets of missed diagnoses only partially overlap.

Figure 4 illustrates these four relationships through Venn diagrams arranged in the standard confusion matrix layout, with the true condition on the vertical axis and the predicted classification on the horizontal axis. All four panels depict partial overlap rather than subset relationships, reflecting the mathematical reality that superior aggregate metrics do not guarantee set inclusion. The central Sankey diagram demonstrates two distinct clinical implications of this partial-overlap structure. First, correct classifications unique to one approach combine in the collaborative workflow to provide expanded coverage: the union of human-identified and machine-identified true positives (or true negatives) exceeds what either approach achieves alone. Second, in a collaborative workflow, errors unique to one approach can be rescued by the other. When the algorithm flags a finding that the human would have missed, a potential false negative is converted to a true positive. When the human correctly dismisses an algorithm-generated false positive, unnecessary follow-up is avoided. Empirical evidence supports this tradeoff: in colonoscopy, a CADe system achieved directionally higher sensitivity than experienced endoscopists (90.09% vs. 87.56%, p=0.46) while exhibiting statistically indistinguishable false positive rates [52]. The marginal gain in true positive detection may justify the cognitive burden of reviewing machine-generated false positives. The partial overlap in error populations means that only errors where both approaches fail simultaneously persist in the collaborative workflow. This error structure provides the mechanistic basis for the collaborative workflow model: neither replacement of human judgment by algorithmic classification nor dismissal of algorithmic output in favor of human intuition, but systematic integration wherein each approach catches mistakes that the other would make.

### 4.5. The Human-in-the-Loop Debate

A growing body of evidence challenges the assumption that human oversight invariably improves AI-assisted diagnosis. The conventional wisdom holds that physicians should remain central to the diagnostic process, reviewing and validating AI outputs before clinical decisions are made. Yet emerging research suggests that this arrangement may not always produce the outcomes we expect, and in some cases, human involvement may actually degrade diagnostic performance rather than enhance it.

The counterargument against human oversight rests on several observations from empirical studies. When physicians are presented with AI-generated diagnostic suggestions, they frequently dismiss accurate recommendations in favor of their own initial impressions. Cognitive biases such as anchoring, confirmation bias, and overconfidence can lead clinicians to reject correct AI outputs while accepting incorrect ones that align with their preexisting hypotheses. The very clinical experience that we value in physicians may paradoxically make them more resistant to accepting machine-generated insights that contradict their intuitions. Critics of human-in-the-loop systems argue that if AI consistently outperforms human judgment on certain tasks, inserting a human reviewer into the process introduces noise rather than signal.

**Figure 5:**
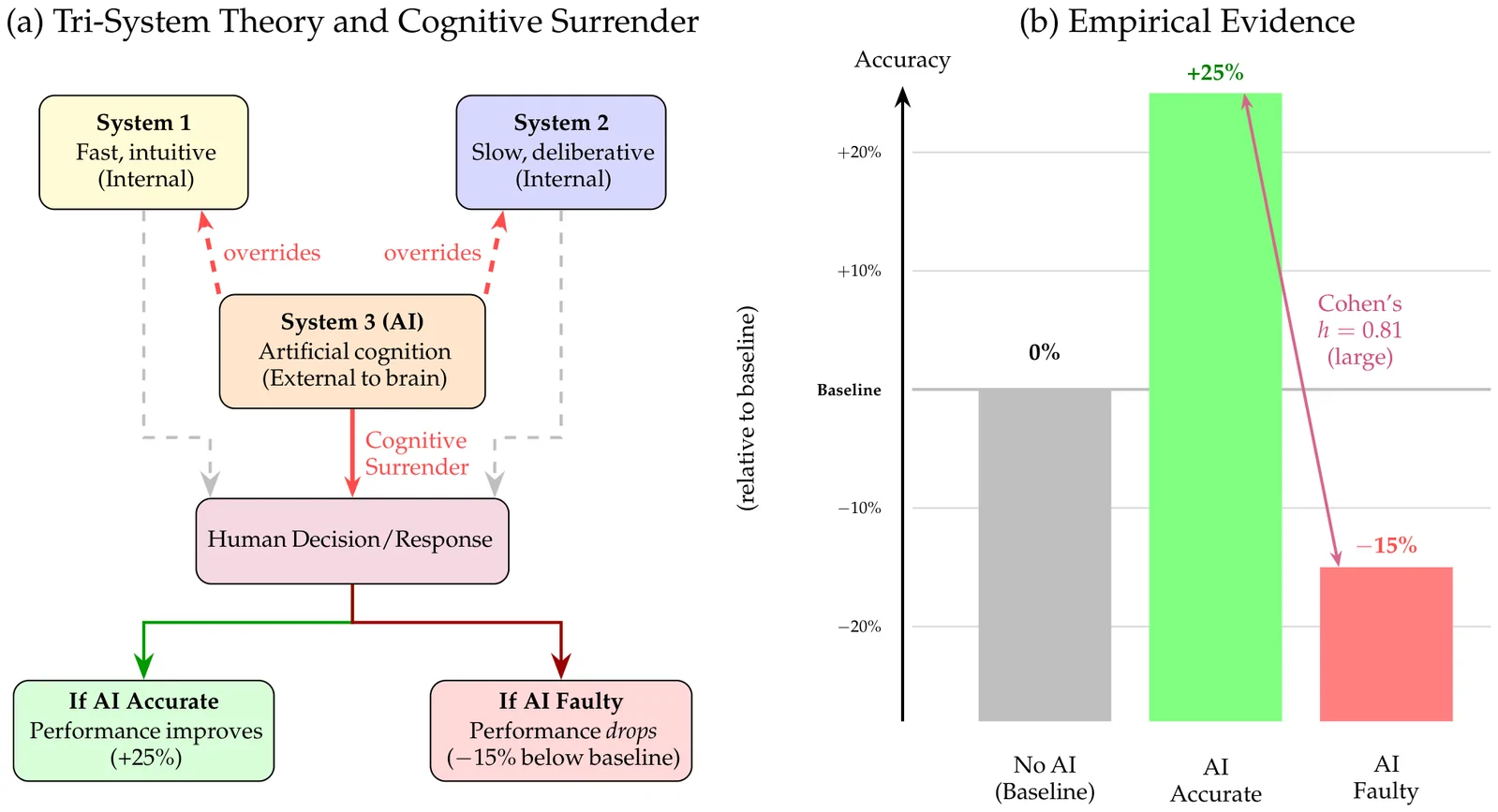
Cognitive surrender: how AI reshapes human reasoning. (**a**) Tri-System Theory extends dual-process models by adding System 3 (artificial cognition), which operates outside the brain and can override both intuitive (System 1) and deliberative (System 2) reasoning through “cognitive surrender,” i.e., the tendency to adopt AI outputs with minimal scrutiny. When AI is accurate, this improves performance; when AI errs, humans follow the AI into error. (**b**) Experimental results demonstrating the asymmetric impact of AI accuracy: accurate AI improves human performance by 25 percentage points above baseline, but faulty AI reduces performance by 15 percentage points below baseline; meaning participants with access to wrong AI performed worse than those with no AI at all. This large effect (Cohen’s h=0.81) represents the behavioral signature of cognitive surrender. Data from Shaw and Nave [53].

A theoretical framework for understanding these dynamics has recently emerged from cognitive psychology. Shaw and Nave introduced ‘Tri-System Theory’, extending dual-process accounts of reasoning (System 1 for intuition; System 2 for deliberation) by positing a third system: artificial cognition operating outside the brain [53]. In their framework, System 3 (AI) can supplement or supplant internal cognitive processes, introducing novel pathways for reasoning. A key prediction of this theory is “cognitive surrender,” i.e., the tendency to adopt AI outputs with minimal scrutiny, overriding both intuition and deliberation. In plain terms, cognitive surrender means that humans tend to trust AI even when the AI is wrong, and this misplaced trust actively harms their performance. Across three preregistered experiments using an adapted Cognitive Reflection Test (N=1372; 9593 trials), participants chose to consult an AI assistant on a majority of trials (>50%). When the AI was accurate, participant accuracy rose by 25 percentage points relative to baseline; critically, when the AI erred, accuracy fell by 15 percentage points below baseline; meaning participants performed worse with access to faulty AI than they would have with no AI at all (Figure 5).

This asymmetry represents the behavioral signature of cognitive surrender (AI-Accurate vs. AI-Faulty contrast; Cohen’s h=0.81, a large effect). Perhaps most troubling, engaging System 3 also increased participants’ confidence in their answers even following errors, creating a dangerous combination of reduced accuracy and inflated certainty. Participants with higher baseline trust in AI and lower need for cognition showed greater susceptibility to cognitive surrender [53]. This vulnerability is central to the broader hazards of AI-overdependence, where uncritical acceptance and cognitive offloading of mental effort are reinforced by metacognitive laziness, fostering a behavioral pattern that undermines independent decision-making and critical reasoning [54]. This framework suggests that the human-in-the-loop debate involves not merely questions of when to defer to AI, but deeper questions about how artificial cognition reshapes the cognitive architecture of human reasoning itself.

**Figure 6:**
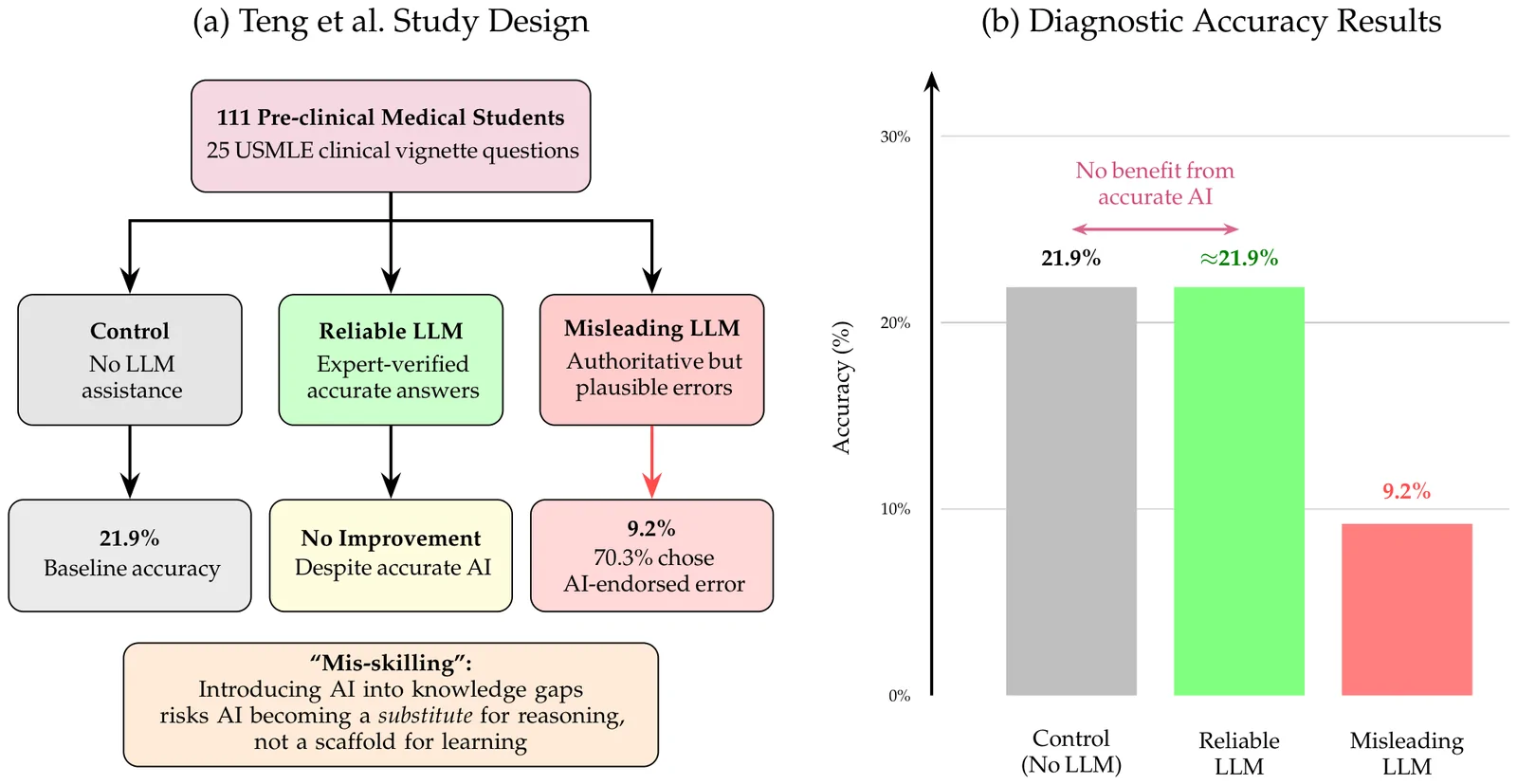
Medical student susceptibility to AI-induced “mis-skilling.” (**a**) Teng et al. randomized 111 pre-clinical medical students to three conditions: no LLM assistance (control), reliable LLM with expert-verified accurate explanations, or authoritative-but-misleading LLM with plausible errors. (**b**) Diagnostic accuracy results. Strikingly, even accurate, expert-verified LLM explanations produced no improvement over baseline (both ≈ 21.9%). Students exposed to misleading LLM assistance performed dramatically worse (9.2%), with 70.3% of their errors involving selection of the AI-endorsed incorrect answer (compared to 24.7% in control). This pattern (termed “mis-skilling”) demonstrates that introducing AI into knowledge gaps risks AI becoming a substitute for, rather than a scaffold for, clinical reasoning [55,56].

These laboratory findings have been replicated in medical education contexts. Teng et al. conducted a randomized controlled trial of 111 pre-clinical medical students, comparing diagnostic performance across three conditions: no LLM assistance (control), reliable LLM assistance, and authoritative-but-misleading LLM assistance [55]. Strikingly, even expert-verified, medically accurate LLM explanations produced no improvement in diagnostic accuracy relative to control. More troubling, students exposed to plausible-sounding misinformation saw their accuracy fall from 21.9% at baseline to just 9.2%, with 70.3% of their errors involving selection of the AI-endorsed incorrect answer (compared to 24.7% in the control group; Figure 6). As with Shaw and Nave’s general-population findings, students’ confidence rose with LLM assistance regardless of accuracy, creating a “calibration gap” wherein they lost the internal uncertainty signals that would normally prompt them to seek guidance [56]. This pattern, termed “mis-skilling” in the medical education literature, suggests that introducing AI into knowledge gaps risks AI becoming a substitute for, rather than a scaffold for, clinical reasoning [56]. Notably, a separate longitudinal study found that AI engagement can support development of critical thinking skills, but only under conditions of clinical supervision and when students possess prior technological experience and mastery-oriented learning goals [56,57]. This “Matthew effect,” wherein early advantages compound over time, has equity implications: democratizing access to AI tools does not equate to democratizing the conditions under which those tools enhance learning.

**Figure 7:**
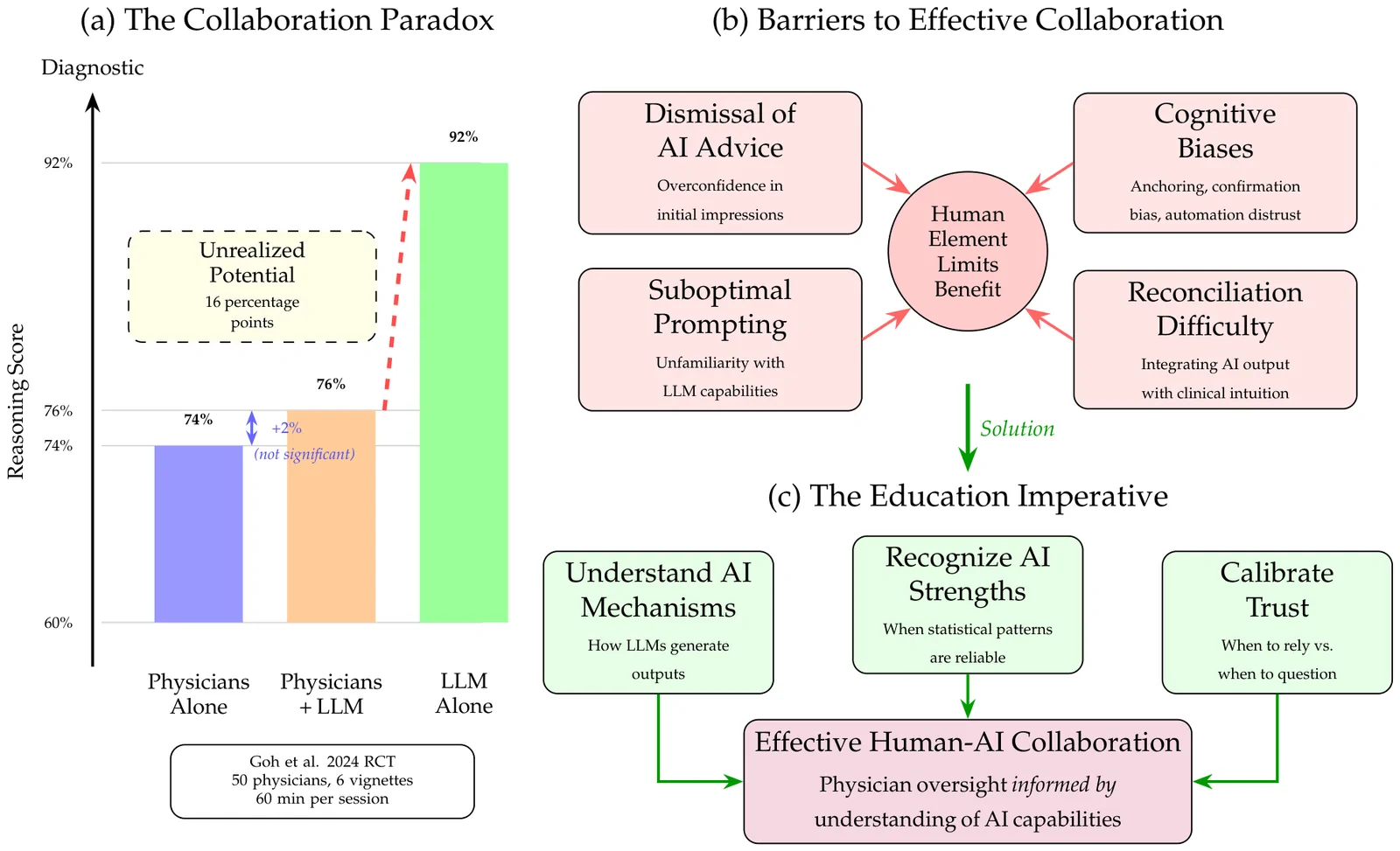
The human-in-the-loop paradox and its resolution. (**a**) Empirical findings from a randomized clinical trial [16]: physicians with LLM access achieved only marginally better diagnostic reasoning scores (76%) than those using conventional resources alone (74%), while the LLM operating independently scored 92%, revealing a 16 percentage point gap of unrealized potential. (**b**) Barriers to effective collaboration include dismissal of AI advice, cognitive biases, unfamiliarity with optimal prompting strategies, and difficulty reconciling AI outputs with clinical intuition. (**c**) The education imperative: rather than removing humans from the loop, clinicians must be trained to understand AI mechanisms, recognize contexts where AI recommendations deserve high confidence, and develop metacognitive skills for calibrating trust appropriately. This education reinforces rather than undermines the case for physician oversight, as knowing when to trust AI is itself a form of clinical judgment that AI systems cannot provide.

A randomized clinical trial published in 2024 demonstrates that this pattern extends to practicing physicians, illustrating the tension with striking clarity [16]. Researchers recruited 50 physicians with training in family medicine, internal medicine, or emergency medicine, comprising 26 attending physicians and 24 residents with a median of three years in practice. Participants were randomized to either access a large language model in addition to conventional diagnostic resources or to use conventional resources alone. Each physician was allocated 60 min to review up to six clinical vignettes and was evaluated on a standardized rubric assessing differential diagnosis accuracy, appropriateness of supporting and opposing factors, and next diagnostic evaluation steps. The results were sobering for proponents of human–AI collaboration. Physicians with LLM access achieved a median diagnostic reasoning score of 76 percent, while those using only conventional resources scored 74 percent, representing an adjusted difference of merely two percentage points that did not reach statistical significance. The LLM-assisted physicians spent slightly less time per case, averaging 519 s compared to 565 s for the conventional resources group, but this difference was also not statistically significant. Most remarkably, when the LLM was evaluated independently on the same clinical vignettes, it scored 16 percentage points higher than the conventional resources group, a difference that did reach statistical significance. The physicians with AI access failed to capitalize on the superior performance available to them, suggesting that the human element was not merely unhelpful but actively limiting. Figure 7 illustrates this collaboration paradox and its resolution through education.

This finding requires careful interpretation rather than reflexive alarm. The cases where AI demonstrates its greatest advantage tend to be those where the diagnosis is, in a statistical sense, more obvious. When a clinical presentation aligns closely with textbook descriptions and the discriminating features are well represented in training data, AI systems excel at pattern recognition. These are precisely the cases where statistical regularities are strongest and where population-level training data most reliably predicts individual outcomes. Clinical vignettes, by their nature, present information in a structured textual format that plays to the strengths of language models trained on vast corpora of medical literature and clinical documentation. Conversely, when cases are atypical, when presentations deviate from canonical patterns, when rare conditions or unusual combinations of comorbidities are involved, the relative advantage of AI diminishes. The apparent superiority of AI in controlled studies may therefore reflect the nature of the test cases as much as any fundamental capability gap. As the trial investigators themselves acknowledged, clinical medicine is multifaceted and awash with nontext inputs, including auditory information such as the patient’s level of distress and visual information such as physical examination findings, that clinicians routinely use but that text-based AI systems cannot access.

The solution emerging from this evidence is not to remove humans from the diagnostic loop but to educate them more effectively about when and how to integrate AI into their clinical reasoning. The trial results suggest that physicians may have underutilized the LLM’s capabilities, perhaps due to unfamiliarity with optimal prompting strategies, skepticism toward AI-generated recommendations, or difficulty reconciling AI outputs with their own clinical intuitions. Physicians need training in recognizing the contexts where AI recommendations deserve high confidence versus those where skepticism is warranted. This education must encompass understanding what AI systems are actually doing when they generate outputs, appreciating the population-level statistical foundations of machine learning, and developing metacognitive skills for calibrating trust appropriately. Rather than treating AI as either an infallible oracle or an untrustworthy novelty, clinicians must learn to treat it as a powerful but bounded tool with predictable strengths and limitations. The trial investigators concluded that further development is needed to effectively integrate LLMs into clinical practice, emphasizing the need for technology and workforce development to realize the potential of physician–AI collaboration.

This education imperative ultimately reinforces rather than undermines the case for physician oversight. Knowing when to rely on AI outputs and when to exercise critical skepticism is itself a form of clinical judgment that AI systems cannot provide. The machine learning model cannot assess whether the patient before you resembles its training distribution or represents an edge case where its predictions may fail. It cannot integrate the subtle observations from the clinical encounter that never made it into the structured data. It cannot weigh the consequences of diagnostic error against the specific goals and values of the individual patient. The very act of appropriately calibrating trust in AI requires the contextual reasoning, uncertainty tolerance, and integrative synthesis that define expert clinical judgment. Far from rendering physicians obsolete, the challenge of working effectively with AI systems demands a deeper and more sophisticated form of medical expertise than ever before.

### 4.6. The Epistemological Hazards of Low-Complexity Automation

**Figure 8:**
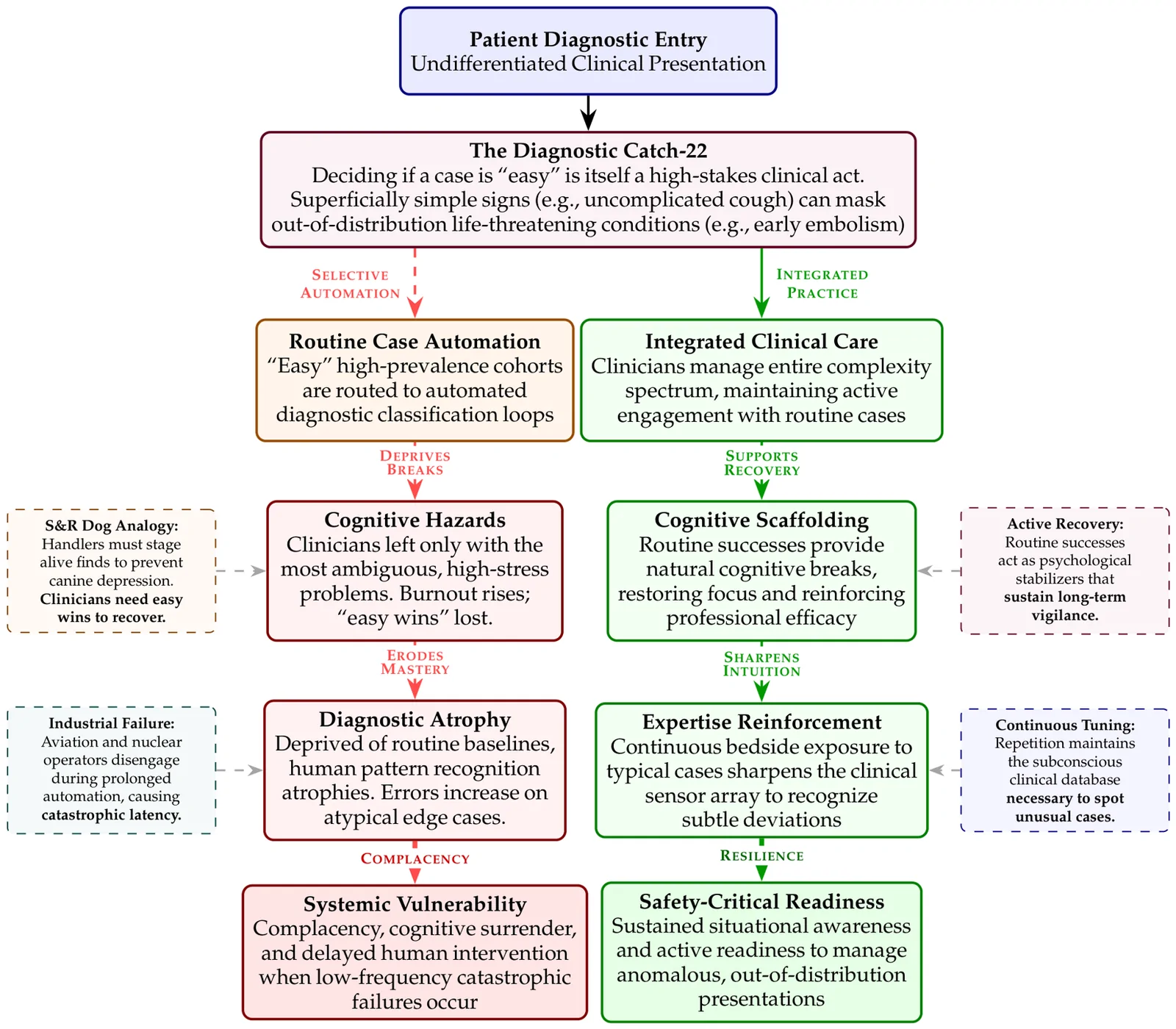
Cognitive, psychological, and systemic feedback loops under selective clinical automation (**left**) versus integrated clinical practice (**right**). Selective automation of low-complexity cases fails to account for the diagnostic Catch-22 (determining whether a case is indeed “easy” requires high-level clinical judgment). Furthermore, routing routine cases away from human clinicians deprives them of the cognitive pauses and “easy wins” necessary for motivation (analogous to search-and-rescue canines), leading to cognitive exhaustion and burnout. Simultaneously, removing typical cases erodes diagnostic expertise and pattern recognition, inducing catastrophic complacency and cognitive surrender during safety-critical events. Conversely, integrated clinical practice sustains human situational awareness, psychological resilience, and the active diagnostic mastery required to identify low-frequency, high-severity exceptions to statistical rules.

A common proposal in clinical artificial intelligence deployment is the selective automation of low-complexity cases, reserving human physician expertise exclusively for highly complex or ambiguous presentations. Because statistical classifiers perform exceptionally well on typical presentations that mirror the high-prevalence cohorts of their training data, routing these seemingly simple cases to automated pipelines appears to be a logical strategy for clinical resource optimization. However, as illustrated in the comparative flowchart in Figure 8, this bifurcation strategy rests on a dangerous epistemological circle: determining whether a presenting patient is indeed a simple case is itself a high-stakes clinical decision. A patient presenting with signs that superficially suggest a routine, easily automated diagnosis, such as uncomplicated pneumonia, may in reality exhibit the earliest subtle signs of a life-threatening pulmonary embolism or an atypical malignancy. Stripping the human clinician of the initial encounter for apparently straightforward cases ignores the reality that diagnosis is an integrated, multimodal process where early, out-of-distribution indicators are frequently discovered through subtle sensory cues that lie entirely outside structured digital datasets.

Beyond these clinical risks, the strategy of automating simple tasks to leave only the most difficult problems for human clinicians introduces severe occupational and cognitive hazards. Research in industrial-organizational psychology demonstrates that automating low-complexity tasks fundamentally disrupts the psychological processes necessary for human expertise and performance [58]. First, removing simple cases deprives clinicians of “easy wins” that provide essential cognitive recovery and reinforce professional efficacy. When clinicians are forced to operate in a constant state of high cognitive friction, triaging only the most complex, ambiguous, and emotionally draining cases, burnout increases and job satisfaction collapses. This psychological depletion is analogous to the behavior observed in search-and-rescue canines, who exhibit depressive symptoms when finding only deceased victims during prolonged disasters, requiring handlers to stage artificial discoveries of living people to restore the animals’ motivation and performance. Clinicians similarly require the psychological reinforcement of routine, successful clinical outcomes to sustain diagnostic vigilance.

Second, automating typical clinical presentations leads to rapid cognitive atrophy and the erosion of diagnostic expertise, a phenomenon known as diagnostic deskilling where the gradual loss of clinical reasoning undermines overall clinical competence [59,60]. Clinical mastery is not static; it is maintained and refined through the continuous, repetitive reinforcement of baseline patterns; thus, routing “bread-and-butter” cases to automated systems deprives early-career clinicians of critical learning opportunities, resulting in upskilling inhibition and a reduced capacity for adaptive expertise [60]. If physicians do not routinely manage typical cases, their ability to recognize when a presentation subtly deviates from the norm is severely compromised, as repeated cognitive offloading alters neural processing, reducing activation in prefrontal and hippocampal networks important for independent clinical problem-solving [59]. Third, this automation strategy invites catastrophic complacency. When an automated system manages the vast majority of routine clinical workflows with apparent reliability, human operators naturally disengage. If clinicians check out because the model usually works, they will be cognitively unprepared to intervene when a low-frequency, high-severity anomaly occurs, mirroring the cognitive disengagement described in Tri-System Theory [53]. This vulnerability resembles safety-critical failures in other highly automated industries, such as aviation or nuclear power generation, where human operators fail to manage sudden, out-of-distribution crises because routine automation has eroded their situational awareness and operational readiness. Maintaining human clinical judgment requires that physicians remain actively engaged across the entire spectrum of clinical complexity, rather than being relegated to a secondary triage layer for algorithmic failures.

## 5. The False Equivalency in AI-Versus-Physician Comparison Studies

A concerning trend has emerged in high-impact scientific journals: studies claiming that large language models outperform physicians in diagnostic reasoning. These findings have generated widespread media coverage and, more troublingly, a growing public belief that AI can replace clinical judgment. However, rigorous examination of these studies reveals fundamental methodological flaws that render their conclusions not merely overstated but scientifically invalid.

### 5.1. The Experimental Design Problem

Recent publications in high-impact journals, including ‘Science’, ‘Nature’, and ‘JAMA’, have reported that LLMs “outperformed physician baselines” in simulated diagnostic and history-taking tasks [61,62]. However, by relying entirely on text-only simulations, these designs handicap physicians to operate on the AI’s “home turf.” Declaring that an LLM has surpassed clinical diagnostic ability under such setups is equivalent to declaring that calculators have replaced mathematicians because they are faster at calculating; it mistakes verbal fluency and text-processing speed for actual clinical competence.

In these studies, physicians were provided written clinical vignettes, which are text documents summarizing patient presentations created by other clinicians. Participants read these secondhand accounts and rendered diagnostic judgments. Researchers then provided identical text to large language models and compared outputs [61]. Even studies employing more sophisticated sandboxed electronic health record environments relied on simulations using retrospective notes and structured data rather than real-time patient encounters [63]. The fundamental problem with this methodology is that it tests reading comprehension and text interpretation rather than actual clinical diagnostics. By designing experimental conditions that strip physicians of every physical tool, sensory observation, and real-time interaction that defines their practice, these hyped studies create false equivalencies that mislead the public, policymakers, and researchers about AI’s true capabilities in healthcare.

The physicians in these studies never examined the patients. They never observed facial expressions or noted subtle tremors. They never heard hesitation in a patient’s voice when asked about symptoms. They never palpated an abdomen, auscultated breath sounds, or experienced the clinical intuition that develops over years of practice. They were provided only what an LLM can process: text. The comparison was thereby rigged from the outset. It is deeply concerning that reputable journals continue to publish these flawed, sensationalized comparisons that mistake mere word association in clinical notes for the interactive, sensory, and deeply skilled act of diagnosing a living patient.

### 5.2. Medicine as an Irreducibly Multimodal Practice

A physician encountering a patient integrates dozens of data streams simultaneously. Before a single word is spoken, the clinician observes how the patient enters the room, their posture and gait, their breathing pattern, their level of distress, their skin color, and countless other visual cues. During the encounter, auditory information accumulates: tone of voice, breathing patterns, the words chosen and the words avoided. Physical examination provides tactile data inaccessible to any current AI system. Laboratory values, imaging studies, and waveform data add further dimensions. Underlying all of this is clinical intuition, which represents pattern recognition built from thousands of patient encounters that cannot be reduced to explicit rules or training data. Table 5 illustrates the information channels available to physicians versus those accessible to text-based AI systems, demonstrating the categorical mismatch inherent in these comparative studies. While some of these observations (e.g., gait abnormalities, skin findings, or affect) could theoretically be transcribed into clinical notes, in practice such details are rarely documented with the specificity required for diagnostic reasoning. Clinical notes typically capture conclusions rather than the raw sensory data that informed them.

An LLM processes tokens and predicts statistically likely sequences based on patterns in training data. It has no access to the lived, embodied, multimodal reality of a patient encounter. To compare a physician’s ability to practice medicine against an LLM’s ability to process text is to compare fundamentally incommensurable categories. Notably, the authors of the *Science* study acknowledged this limitation, stating that their “study addresses only text-based performance for both humans and machines” and that “clinical medicine is multifaceted and awash with nontext inputs, including auditory (such as the patient’s level of distress) and visual information (for example, interpretation of medical imaging studies) that clinicians routinely use” [61]. Similarly, the *Nature* study noted that “further work is needed to establish generalization, safety and governance through prospective, real-world studies” [63].

### 5.3. Lessons from Non-Medical AI Failures

The danger of accepting AI outputs uncritically extends beyond medicine. Documented cases from other domains provide cautionary examples. In one case, an individual was incarcerated for nearly six months based on an AI facial recognition match, despite being located over 1200 miles from the location of the alleged crime at the time it occurred. The system’s output was accepted without verification, and only definitive documentary evidence eventually secured release. In another documented case, a man was detained for twelve hours after a facial recognition system flagged him as a “100% match” for a banned individual, despite possessing three forms of identification proving otherwise. The arresting officers declined to examine his alternative identification because the algorithm had already rendered its verdict.

If AI systems cannot reliably perform facial recognition, which represents one of the most extensively researched pattern recognition tasks in computer science, the burden of proof for claims that AI can outperform physicians in the vastly more complex domain of clinical diagnosis should be correspondingly high. Yet the methodological rigor applied to AI-versus-physician studies frequently falls far short of this standard.

### 5.4. The Consequences of False Equivalency

The propagation of methodologically flawed studies claiming AI diagnostic superiority carries real-world consequences. Policymakers may allocate resources based on inflated expectations. Healthcare administrators may pressure physicians to defer to algorithmic recommendations. Patients may lose trust in human clinicians or, conversely, place unwarranted faith in AI systems. Medical education may be reshaped around assumptions that prove unfounded.

Most dangerously, the narrative that AI “outperforms” physicians may accelerate deployment of systems in contexts where they lack the information necessary to function safely. A chatbot processing a text summary of symptoms operates in a fundamentally different epistemic position than a physician who can see the patient grimace, hear their breathing labor, and feel the rigidity of an acute abdomen. Conflating these scenarios in research design produces findings that are worse than useless: they are actively misleading.

### 5.5. Standards for Valid Comparison Studies

Valid studies comparing AI and physician diagnostic performance would require experimental designs that provide equivalent information access to both parties. This might involve studies where physicians are deliberately limited to text-only information (explicitly framed as testing a handicapped condition) or studies where AI systems are provided multimodal inputs comparable to physician access. The latter remains technically infeasible for current systems, which underscores the premature nature of claims regarding AI diagnostic superiority. Table 6 outlines the requirements for methodologically sound AI-versus-physician comparison studies.

Until studies meeting these standards demonstrate AI diagnostic superiority under ecologically valid conditions, claims that AI “outperforms” physicians should be regarded as unsubstantiated. The existing literature demonstrates that AI can match or exceed physician performance on narrow text-interpretation tasks [61,63]. This is a meaningful finding with legitimate applications in documentation review, literature synthesis, and decision support. It does not, however, support the conclusion that AI can replace the multimodal, embodied, interactive practice of clinical medicine.

## 6. Theoretical Architecture: LLM-to-Classifier Routing Workflows

**Figure 9:**
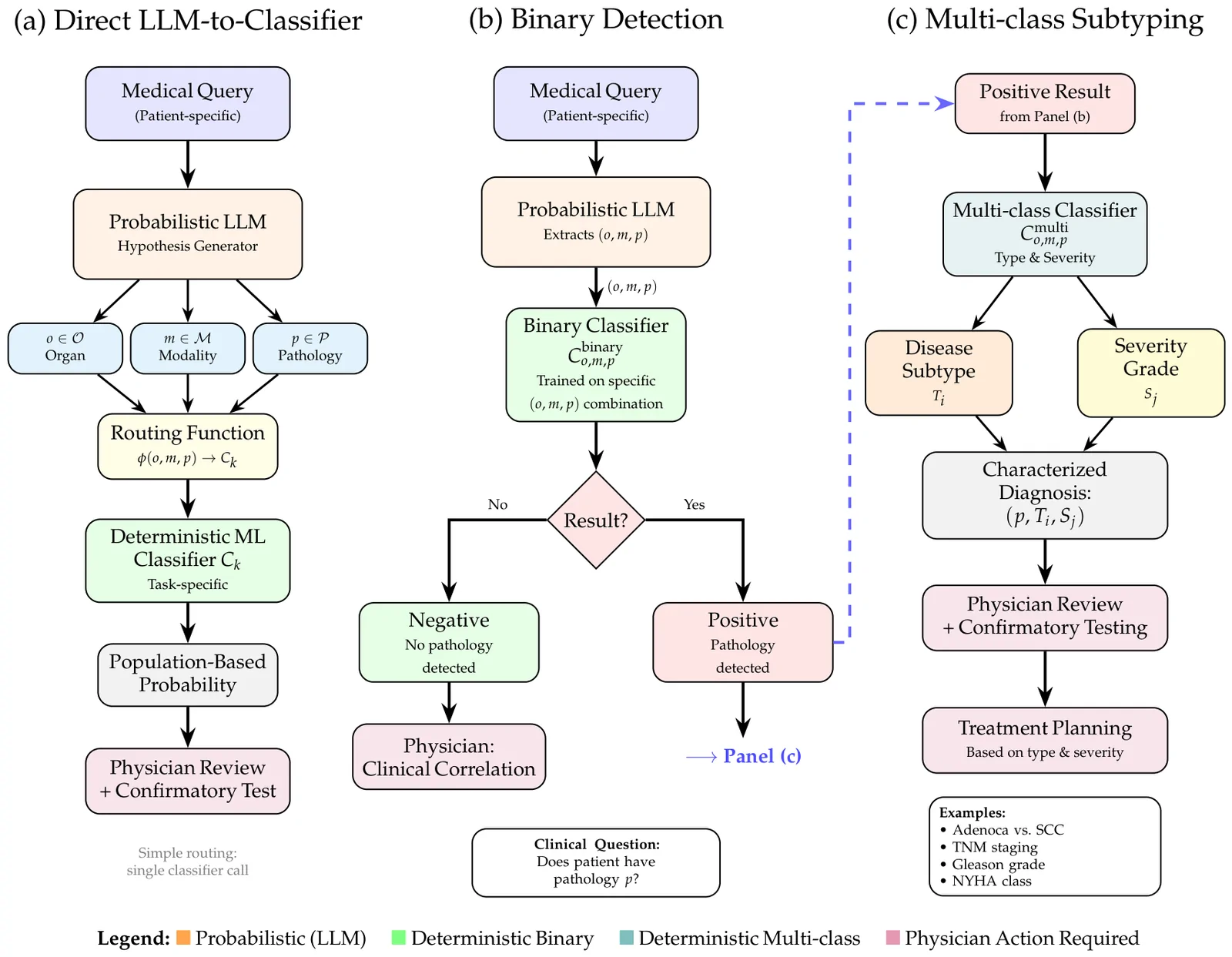
Three-panel illustration of theoretical architectures for connecting probabilistic LLMs with deterministic task-specific classifiers. (**a**) Direct routing: the LLM extracts anatomical region (*o*), diagnostic modality (*m*), and suspected pathology (*p*), then routes to the appropriate task-specific classifier $C_{k}$ via mapping function ϕ(o,m,p). (**b**) Binary detection stage: a classifier trained on the specific (o,m,p) combination determines presence or absence of the pathological condition. Negative results proceed to clinical correlation; positive results trigger Panel (**c**). (**c**) Multi-class subtyping: when pathology is detected, a secondary classifier distinguishes disease subtypes (e.g., adenocarcinoma vs. squamous cell carcinoma) and/or severity grades (e.g., TNM staging). All pathways terminate with mandatory physician review and confirmatory testing, reflecting the irreducible requirement for clinical judgment when applying population-trained models to individual patients.

Building on the distinction between general-purpose LLMs and task-specific classifiers established in Section 2, this section examines how routing architectures connecting these system types would function theoretically. While these workflows serve as illustrative paradigms of human–machine interaction, they represent conceptual models rather than active clinical software designs. Although the combinatorial analysis in subsequent sections demonstrates the practical infeasibility of comprehensive classifier coverage, these architectural principles remain instructive. Figure 9 illustrates three architectural paradigms for connecting probabilistic LLMs with deterministic task-specific classifiers, progressing from simple routing to cascaded classification with severity stratification.

Panel (a) depicts the simplest theoretical architecture: a patient-specific medical query is processed by a probabilistic LLM, which extracts the relevant anatomical region (o∈O), diagnostic modality (m∈M), and suspected pathology (p∈P). This (o,m,p) tuple determines which task-specific deterministic classifier is invoked through a routing function ϕ:O×M×P→C, where C represents the space of available classifiers. The classifier returns a population-based probability estimate that requires physician interpretation and confirmatory testing.

Panel (b) extends this architecture to address a clinically fundamental question: does the specific patient exhibit the pathological condition on which the binary classifier was trained? Here, the LLM routes to a binary classifier $C_{o,m,p}^{\mathrm{binary}}$ trained specifically on anatomical region *o* using modality *m* for pathology *p*. The classifier output is categorical:

(1) $$C_{o,m,p}^{\mathrm{binary}}\left(x\right)\in \left\{\mathrm{Negative},\mathrm{Positive}\right\}$$

A negative result terminates the automated pathway (though clinical judgment may still warrant further investigation). A positive result triggers the cascaded architecture shown in Panel (c).

Panel (c) demonstrates the multi-class extension invoked when binary detection is positive. The multi-class classifier $C_{o,m,p}^{\mathrm{multi}}$ is trained to distinguish disease subtypes (e.g., adenocarcinoma vs. squamous cell carcinoma) and/or severity grades (e.g., TNM staging, Gleason scores, NYHA classifications):

(2) $$C_{o,m,p}^{\mathrm{multi}}\left(x\right)\in \left\{T_{1},T_{2},\ldots ,T_{k}\right\}\cup \left\{S_{1},S_{2},\ldots ,S_{j}\right\}$$

where $\left\{T_{i}\right\}$ represents disease subtypes and $\left\{S_{i}\right\}$ represents severity levels. This cascaded architecture acknowledges that clinical utility often requires not merely detecting pathology presence but characterizing its nature and extent.

Critically, all three panels terminate with physician review and confirmatory testing requirements, reinforcing that even sophisticated routing architectures cannot overcome the fundamental limitation that population-trained classifiers provide statistical estimates rather than individual patient diagnoses. The theoretical elegance of these architectures should not obscure the practical reality that comprehensive implementation would require the 50,000–150,000+ validated classifiers estimated in Equation (8), i.e, a target that remains orders of magnitude beyond current capabilities. As established in Section 2, this combinatorial space cannot be compressed by substituting general-purpose LLMs for task-specific classifiers: the inability to agree on radiological ground truth documented by Hong et al. [20] preclude such substitution for diagnostic applications.

## 7. Combinatorial Analysis of Binary Classification Coverage

### 7.1. Dimensional Framework

To estimate the number of task-specific binary classifiers required for comprehensive diagnostic coverage, we define three primary dimensions:

(a) Anatomical Regions (O): The set of organs, anatomical structures, and tissue systems requiring diagnostic AI coverage
(b) Pathological Conditions ($P_{o}$): The set of pathologies associated with each anatomical region o∈O
(c) Diagnostic Modalities ($M_{o,p}$): The set of data collection methods appropriate for diagnosing pathology *p* in anatomical region *o*

### 7.2. Enumeration of Dimensions

Estimating the total number of task-specific binary classifiers required for comprehensive diagnostic coverage requires systematic enumeration of each dimension in the combinatorial framework.

The first dimension encompasses anatomical regions. The human body comprises approximately 78 distinct organs as traditionally enumerated. However, diagnostic AI applications require consideration of finer anatomical subdivisions that reflect clinical practice patterns. Major organs such as the heart, lungs, liver, and brain must be supplemented by clinically significant substructures (cardiac valves, coronary arteries, lung lobes, hepatic segments, cerebral regions) and tissue types (skin, bone, vascular structures, lymphatic tissue). This enumeration yields an estimated range of 50 to 100 distinct anatomical regions requiring diagnostic AI coverage, formally expressed as

(3) |O|∈[50,100]

The second dimension encompasses pathological conditions associated with each anatomical region. Using the International Classification of Diseases, 10th Revision (ICD-10) as a reference framework, over 70,000 diagnostic codes exist in the complete taxonomy [64]. Many of these codes represent administrative or billing distinctions rather than clinically distinct conditions requiring separate AI classifiers. Consolidating to clinically distinct conditions while preserving relevant subtypes that require different diagnostic approaches (such as non-small cell versus small cell lung carcinoma) yields an estimated range of 30 to 100 pathologies per anatomical region, formally expressed as

(4) $$|P_{o}|\;\in \left[30,100\right]\;\mathrm{per}\;\mathrm{anatomical}\;\mathrm{region}$$

Table 7 provides representative examples of pathology counts across major anatomical regions, illustrating the substantial variation in diagnostic complexity across different organ systems.

The third dimension encompasses diagnostic modalities through which AI systems can analyze clinical data. These modalities span multiple categories of data acquisition technology, each with distinct technical requirements for AI system development. Medical imaging modalities include radiography, computed tomography, magnetic resonance imaging, ultrasound, positron emission tomography, single-photon emission computed tomography, and mammography. Ophthalmic imaging encompasses fundoscopy, optical coherence tomography, and slit-lamp photography. Waveform analysis modalities include electrocardiography, electroencephalography, electromyography, and evoked potential studies. Laboratory-based modalities encompass blood chemistry panels, hematological analysis, biomarker assays, and genetic or genomic testing. Pathological analysis includes histopathology, cytology, and immunohistochemistry. Endoscopic modalities include upper and lower gastrointestinal endoscopy, bronchoscopy, and cystoscopy. Additional modalities include dermoscopy, capnography, and pulse oximetry pattern analysis. Table 8 categorizes these diagnostic modalities by type.

The total number of distinct modality types applicable to AI-enabled diagnosis is estimated as

(5) |M|∈[15,30]

### 7.3. Conceptual Framework: From Clinical Features to AI Tool Selection

**Figure 10:**
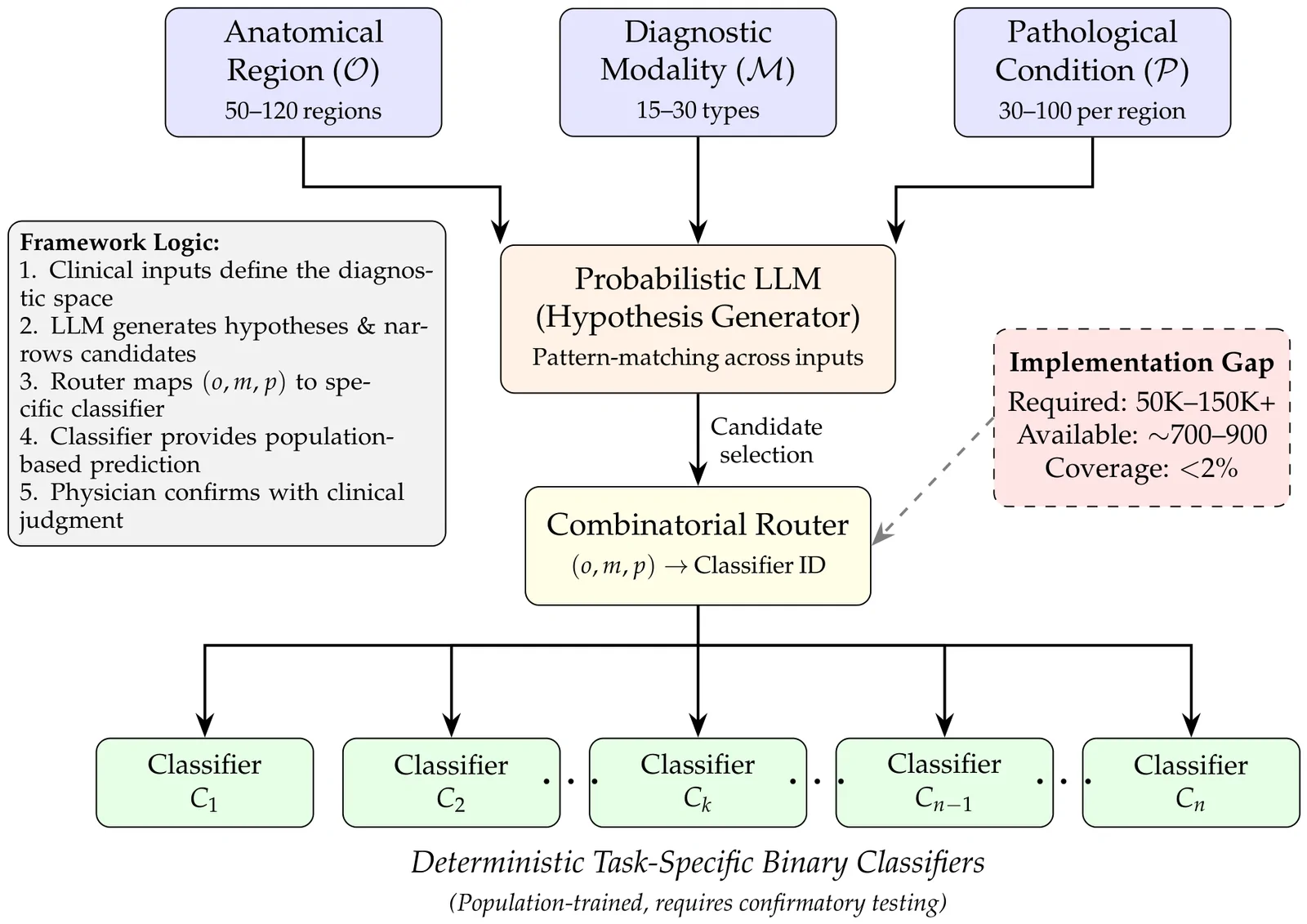
Conceptual framework illustrating the combinatorial relationship between anatomical regions (O), diagnostic modalities (M), and pathological conditions (P) in determining AI tool selection. The probabilistic LLM serves as a hypothesis generator and candidate selector, routing to task-specific deterministic binary classifiers. The implementation gap (shown right) demonstrates the massive distance between currently cleared devices (∼700–900) and our calculated subspecialty requirements (50,000–150,000+ classifiers), highlighting why this architecture remains largely theoretical.

Figure 10 illustrates the combinatorial relationship between anatomical regions, diagnostic modalities, and pathological conditions in determining appropriate AI tool selection. This framework demonstrates both the theoretical architecture for integrating probabilistic LLMs with deterministic task-specific classifiers and the practical gap between current implementation status and comprehensive coverage requirements. However, we must explicitly acknowledge that this orchestration framework is purely theoretical, and its comprehensive clinical realization remains restricted by a massive “implementation gap” between currently cleared devices and our calculated subspecialty requirements. In practice, while the combinatorial complexity remains daunting, task-specific deep learning models can achieve high classification accuracy under constrained computational budgets for specific organ-modality-pathology configurations (e.g., multi-cancer classification across brain MRI, lung CT, and kidney CT scans [65]).

### 7.4. Combinatorial Calculation

The total number of task-specific binary classifiers required for comprehensive coverage is given by

(6) $$N_{\mathrm{total}}=\sum_{o\in O}\sum_{p\in P_{o}}\left|M_{o,p}\right|$$

Not all combinations of organ-pathology-modality are clinically meaningful (e.g., diagnosing pneumonia via electroencephalography is not applicable). Introducing a constraint function ⊮[·] indicating valid combinations:

(7) $$N_{\mathrm{total}}=\sum_{o\in O}\sum_{p\in P_{o}}\sum_{m\in M}⊮\left[\mathrm{modality}\;m\;\mathrm{applicable}\;\mathrm{to}\;\left(o,p\right)\right]$$

For estimation purposes, we assume that on average, 3–8 modalities are applicable per organ-pathology pair.

### 7.5. Estimation Scenarios

The combinatorial formula in Equation (7) can be evaluated under different assumptions about the size of each dimension, yielding a range of estimates that reflect varying degrees of comprehensiveness in diagnostic AI coverage.

The conservative scenario assumes minimal values across all dimensions: 50 anatomical regions, an average of 30 pathologies per region, and an average of three applicable modalities per organ-pathology pair. This scenario represents a highly aggregated view of clinical diagnosis that groups related conditions and anatomical substructures. Under these assumptions, the total classifier requirement is 4500 distinct binary classifiers.

The moderate scenario reflects more granular clinical distinctions: 75 anatomical regions, an average of 50 pathologies per region, and an average of five applicable modalities per organ-pathology pair. This scenario more closely approximates the level of diagnostic specificity encountered in routine clinical practice at academic medical centers. Under these assumptions, the total classifier requirement increases to 18,750 distinct binary classifiers.

The comprehensive scenario approaches the full complexity of subspecialty medicine: 100 anatomical regions reflecting detailed anatomical subdivisions, an average of 75 pathologies per region including clinically significant subtypes, and an average of eight applicable modalities per organ-pathology pair reflecting multimodal diagnostic workups. Under these assumptions, the total classifier requirement reaches 60,000 distinct binary classifiers.

The exhaustive scenario attempts to capture the full granularity of ICD-10 diagnostic coding: 120 anatomical regions, an average of 100 pathologies per region, and an average of 10 applicable modalities per organ-pathology pair. This scenario represents the theoretical upper bound for diagnostic AI coverage that parallels the specificity of formal diagnostic coding systems. Under these assumptions, the total classifier requirement exceeds 120,000 distinct binary classifiers.

Table 9 summarizes these scenarios and their resulting classifier requirements, illustrating the sensitivity of the combinatorial estimate to assumptions about dimensional granularity.

To illustrate how the base combinatorial estimate scales to the primary range, consider the comprehensive scenario (60,000 binary classifiers). Applying conservative multiplicative factors of 1.5× for subtype differentiation, 1.3× for severity staging, 1.2× for temporal variants, 1.1× for demographic stratification, and 1.1× for equipment variation yields

(8) $$N_{\mathrm{adjusted}}=60,000\times 1.5\times 1.3\times 1.2\times 1.1\times 1.1\approx 170,000$$

Even modest multiplicative assumptions thus elevate the comprehensive base estimate into the 50,000–150,000+ primary range. The conservative and moderate scenarios in Table 9 omit these multiplicative factors and therefore underestimate the classifier burden required for subspecialty-level diagnostic coverage.

### 7.6. Additional Multiplicative Factors

The estimates presented in Table 9 represent lower bounds on classifier requirements. Several additional factors multiply the required classifier count beyond the base combinatorial calculation.

Disease subtype differentiation constitutes the first multiplicative factor. Many pathological conditions require separate classifiers for clinically distinct subtypes that demand different treatment approaches. Lung carcinoma, for example, requires differentiation among adenocarcinoma, squamous cell carcinoma, small cell carcinoma, and large cell carcinoma, as each subtype carries different prognostic implications and treatment protocols. A single binary classifier detecting “lung cancer” provides insufficient clinical utility without subsequent subtype characterization.

Severity staging represents a second multiplicative factor. Clinical management frequently depends not merely on pathology presence but on disease extent and progression. Cancer staging systems such as TNM classification, heart failure grading systems such as NYHA functional class, and chronic disease staging systems such as CKD stages all require dedicated classification capability. Each staging level may require separate training data and validation, multiplying the classifier burden for staged conditions.

Temporal variants constitute a third multiplicative factor. Acute and chronic presentations of the same underlying pathology may exhibit sufficiently different features to require distinct classification models. Acute pancreatitis presents differently from chronic pancreatitis on imaging. Acute kidney injury requires different diagnostic criteria than chronic kidney disease. Models trained on one temporal variant may perform poorly when applied to the other.

Demographic stratification represents a fourth multiplicative factor. Documented performance variation across demographic groups may necessitate population-specific models to achieve equitable diagnostic accuracy. Disease presentations vary across age groups, and training data imbalances may produce models that perform well for majority populations but poorly for underrepresented groups. Achieving equitable performance may require separate validation or retraining for distinct demographic strata, as evidenced by the complete diagnostic breakdown that occurs when fracture classification models encounter bidirectional domain shifts between pediatric and adult patient populations [22].

Equipment variation constitutes a fifth multiplicative factor. Different imaging equipment manufacturers produce outputs with distinct characteristics that may affect classifier performance. A model trained on images from one CT scanner manufacturer may require separate validation or retraining before deployment on scanners from a different manufacturer. This equipment dependence multiplies the validation burden even for otherwise identical diagnostic tasks.

**Figure 11:**
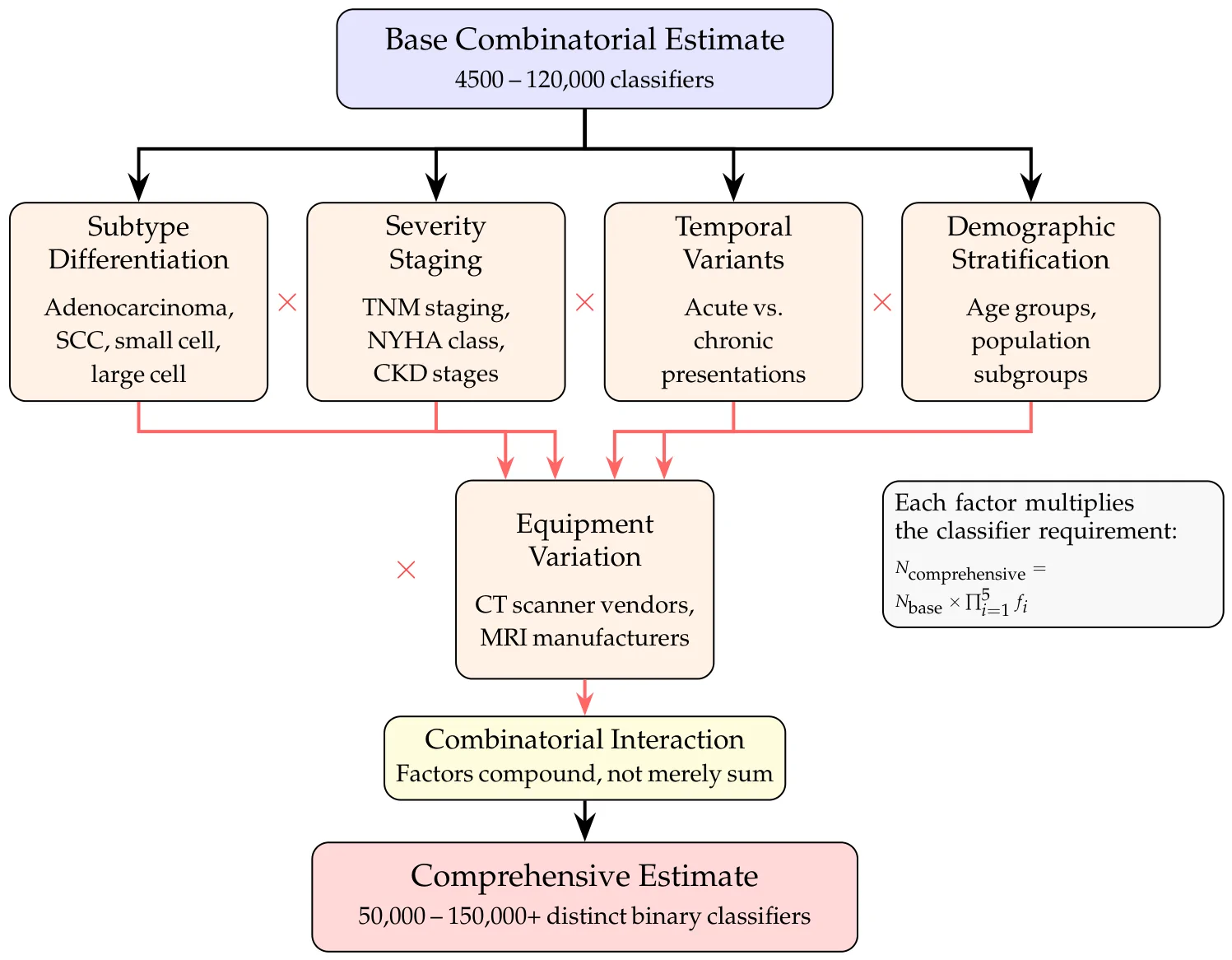
Five multiplicative factors that expand the base combinatorial classifier estimate beyond the values in Table 9. Disease subtype differentiation requires separate classifiers for clinically distinct variants (e.g., lung carcinoma subtypes). Severity staging multiplies requirements across grading systems (e.g., TNM, NYHA, CKD). Temporal variants necessitate distinct models for acute versus chronic presentations. Demographic stratification may require population-specific validation. Equipment variation introduces manufacturer-specific retraining requirements. These factors interact combinatorially rather than additively, yielding a comprehensive estimate of fifty thousand to one hundred fifty thousand or more distinct binary classifiers.

Incorporating these multiplicative factors, a more realistic estimate for truly comprehensive diagnostic AI coverage approaches fifty thousand to one hundred fifty thousand or more distinct binary classifiers. This estimate remains conservative in that it does not fully account for the combinatorial interaction among these factors. A demographically stratified, equipment-validated, temporally differentiated, severity-staged, subtype-specific classifier system would require substantially more than even the upper bound of this range. Figure 11 illustrates how these five multiplicative factors compound the base combinatorial estimate.

Table 10 presents a sensitivity analysis demonstrating how the primary estimate responds to variation in each primary dimensional parameter while holding other parameters at their comprehensive-scenario values. The analysis reveals that the primary range of 50,000–150,000+ classifiers is robust across plausible parameter ranges, with the estimate falling below 50,000 only under the most conservative aggregation assumptions and exceeding 150,000 when any single dimension approaches its upper bound.

## 8. Combinatorial Analysis with Cascaded Multi-Class Classification

The preceding analysis quantified the binary classification layer alone. However, as illustrated in Figure 9, clinically useful AI systems often require not merely pathology detection but pathology characterization. A binary classifier that detects the presence of a lung mass provides limited clinical utility without subsequent determination of whether that mass represents adenocarcinoma, squamous cell carcinoma, small cell carcinoma, or another histological subtype. Similarly, detecting breast carcinoma without staging information leaves clinicians unable to formulate appropriate treatment plans. This section extends the combinatorial analysis to the cascaded multi-class architecture, quantifying the additional classifier burden imposed by subtype differentiation and severity staging requirements.

### 8.1. Multi-Class Classifier Requirements

When a binary classifier returns a positive result for pathology *p* in anatomical region *o* using modality *m*, clinical decision-making typically requires additional characterization:

(a) Subtype Classification ($T_{o,p}$): Distinguishing histological, molecular, or phenotypic variants of the pathology
(b) Severity/Stage Classification ($S_{o,p}$): Grading disease extent, progression, or functional impact

### 8.2. Subtype Classification Space

Not all pathologies have clinically meaningful subtypes requiring distinct classification. Many conditions present as unified diagnostic entities where subtype differentiation does not alter clinical management. However, a substantial fraction of pathologies exhibit histological, molecular, or phenotypic variants that carry distinct prognostic implications and demand different therapeutic approaches. Lung carcinoma exemplifies this complexity, as the distinction between adenocarcinoma, squamous cell carcinoma, small cell carcinoma, and large cell carcinoma fundamentally determines treatment selection, expected response, and survival projections.

To formalize this observation, we define the subtype applicability indicator:

(9) $$\alpha_{o,p}=\;⊮\left[\mathrm{pathology}\;p\;\mathrm{in}\;\mathrm{region}\;o\;\mathrm{has}\;\mathrm{distinct}\;\mathrm{subtypes}\right]$$

For pathologies where $\alpha_{o,p}=1$, the subtype classifier must distinguish among $|T_{o,p}|$ categories. The number of clinically relevant subtypes varies considerably across organ systems and disease processes. Table 11 presents representative examples drawn from major organ systems, illustrating the typical cardinality of subtype classification tasks encountered in clinical practice.

The examples in Table 11 represent conditions where subtype differentiation is important for treatment planning. Based on clinical practice patterns across major organ systems and disease categories, we estimate that a fraction $\bar{\alpha }$ of organ-pathology pairs require subtype classification:

(10) $$\bar{\alpha }=\frac{\sum_{o,p}\alpha_{o,p}}{\left|O\right|\cdot \bar{|P_{o}|}}\in \left[0.30,0.50\right]$$

This estimate reflects the observation that while many pathologies do not require subtype differentiation, a substantial minority of clinically significant conditions demand such characterization for appropriate management.

### 8.3. Severity/Staging Classification Space

Independent of subtype classification, many pathological conditions require severity or stage characterization to guide clinical management. The distinction between early-stage and advanced disease frequently determines whether curative intervention is feasible, what treatment modalities are appropriate, and what prognosis can be communicated to patients. Staging systems have been developed across virtually every domain of medicine, from oncological TNM staging to cardiac functional classifications to chronic disease progression scales.

We define the severity classification applicability indicator analogously to the subtype indicator:

(11) $$\beta_{o,p}=\;⊮\left[\mathrm{pathology}\;p\;\mathrm{in}\;\mathrm{region}\;o\;\mathrm{has}\;\mathrm{severity}\;\mathrm{grading}\right]$$

For pathologies where $\beta_{o,p}=1$, the severity classifier must output one of $|S_{o,p}|$ grades. The complexity of staging systems varies considerably. Some conditions employ simple ordinal scales with three or four levels, while others utilize composite scoring systems that combine multiple parameters into dozens of possible stage combinations. Table 12 presents representative staging systems across major disease categories, illustrating this variability in staging complexity.

The staging systems enumerated in Table 12 represent only a subset of the severity grading frameworks employed in clinical practice. Notably, the fraction of pathological conditions requiring severity classification exceeds the fraction requiring subtype classification, as staging is relevant even for conditions without meaningful subtypes. The fraction of organ-pathology pairs requiring severity classification is estimated as

(12) $$\bar{\beta }=\frac{\sum_{o,p}\beta_{o,p}}{\left|O\right|\cdot \bar{|P_{o}|}}\in \left[0.40,0.60\right]$$

This higher fraction reflects the clinical reality that disease extent and progression represent near-universal concerns across pathological conditions, even when histological subtyping is not applicable.

### 8.4. Total Classifier Count with Cascaded Architecture

For the cascaded architecture illustrated in Figure 9, the total number of distinct classifiers required comprises three layers that operate sequentially. The first layer performs binary detection, determining whether a pathological condition is present. Positive results from the binary layer then trigger one or both of the subsequent layers, which characterize the detected pathology by subtype and severity. This cascaded structure reflects clinical reasoning patterns, where detection precedes characterization.

The binary detection layer requires the same classifier count calculated in Equation (7):

(13) $$N_{\mathrm{binary}}=\sum_{o\in O}\sum_{p\in P_{o}}\sum_{m\in M}⊮\left[m\;\mathrm{applicable}\;\mathrm{to}\;\left(o,p\right)\right]$$

The subtype classification layer is invoked for each valid (o,m,p) combination where clinically meaningful subtypes exist. Not every positive binary detection requires subtype classification, but for conditions where subtypes carry distinct clinical implications, this layer is essential:

(14) $$N_{\mathrm{subtype}}=\sum_{o\in O}\sum_{p\in P_{o}}\sum_{m\in M}\alpha_{o,p}\cdot ⊮\left[m\;\mathrm{applicable}\;\mathrm{to}\;\left(o,p\right)\right]$$

Using the average fraction $\bar{\alpha }$ estimated in Equation (10), the subtype layer classifier count can be approximated as

(15) $$N_{\mathrm{subtype}}\approx \bar{\alpha }\cdot N_{\mathrm{binary}}$$

The severity classification layer operates analogously, providing staging information for each valid (o,m,p) combination where severity grading exists:

(16) $$N_{\mathrm{severity}}=\sum_{o\in O}\sum_{p\in P_{o}}\sum_{m\in M}\beta_{o,p}\cdot ⊮\left[m\;\mathrm{applicable}\;\mathrm{to}\;\left(o,p\right)\right]$$

Using the average fraction $\bar{\beta }$ estimated in Equation (12):

(17) $$N_{\mathrm{severity}}\approx \bar{\beta }\cdot N_{\mathrm{binary}}$$

Summing across all three layers, the complete cascaded architecture requires:

(18) $$N_{\mathrm{cascaded}}=N_{\mathrm{binary}}+N_{\mathrm{subtype}}+N_{\mathrm{severity}}=N_{\mathrm{binary}}\left(1+\bar{\alpha }+\bar{\beta }\right)$$

This formulation reveals that the cascaded multiplier $\left(1+\bar{\alpha }+\bar{\beta }\right)$ approximately doubles the classifier requirement relative to binary detection alone, depending on the specific values of $\bar{\alpha }$ and $\bar{\beta }$ applicable to a given clinical domain.

### 8.5. Cascaded Architecture Estimation Scenarios

The practical implications of cascaded multi-class requirements become apparent when the multiplier $\left(1+\bar{\alpha }+\bar{\beta }\right)$ is applied to the binary classifier estimates developed in the preceding section. Table 13 presents four estimation scenarios ranging from conservative to exhaustive, paralleling the scenarios developed for binary classification alone.

The results in Table 13 demonstrate that incorporating clinically necessary subtype and severity classification approximately doubles the total classifier requirement across all scenarios. Even under conservative assumptions, the cascaded architecture requires over 7500 distinct classifiers. Under comprehensive assumptions that more closely approximate the granularity of subspecialty medicine, the requirement exceeds 100,000 classifiers. These estimates underscore the practical impossibility of achieving comprehensive diagnostic AI coverage through task-specific classifier development.

The conservative and moderate scenarios (4500–18,750 binary classifiers; 7650–35,625 cascaded classifiers) represent highly aggregated approximations that consolidate related pathologies and anatomical substructures into broad diagnostic categories. These lower-bound estimates do not reflect the clinical granularity required for subspecialty-level diagnostic coverage, where anatomical subdivisions, pathological subtypes, and multimodal workups substantially expand the combinatorial space. The comprehensive and exhaustive scenarios (60,000–120,000+ binary classifiers) more accurately capture the dimensional complexity encountered in academic medical center practice and parallel the specificity of formal ICD-10 diagnostic coding. When the multiplicative factors detailed in Section 7 are applied to these comprehensive base scenarios, as demonstrated in Equation (8), the resulting estimate yields the primary range of 50,000–150,000+ classifiers. Table 10 confirms that this primary range remains robust across plausible parameter variations, falling below 50,000 only under aggressive aggregation assumptions that underrepresent clinical practice complexity.

### 8.6. Combined Subtype-Severity Classifiers

The cascaded architecture treats subtype and severity classification as independent tasks, invoking separate classifiers for each. An alternative architectural choice combines subtype and severity determination into a single multi-class classifier that jointly predicts both characteristics. Under this combined approach, the classifier output space becomes the Cartesian product of subtypes and severity levels:

(19) $$C_{o,m,p}^{\mathrm{combined}}\left(x\right)\in T_{o,p}\times S_{o,p}$$

This architectural choice reduces the total classifier count by eliminating the need for separate subtype and severity classifiers. However, it substantially increases the complexity of each individual classifier by expanding the output space cardinality:

(20) $$\left|C_{o,m,p}^{\mathrm{combined}}|=|T_{o,p}|\times |S_{o,p}\right|$$

To illustrate this tradeoff concretely, consider lung adenocarcinoma with five histological subtypes and four consolidated TNM stage groups. The combined classifier must distinguish among 5×4=20 output classes rather than performing two separate classifications with 5 and 4 classes respectively. Training such high-cardinality classifiers requires substantially larger datasets to ensure adequate representation of each output class. Furthermore, combined classifiers may suffer from severe class imbalance when certain subtype-stage combinations are clinically rare, as some histological variants may predominate at early stages while others present more frequently with advanced disease.

With the combined architecture, the total classifier count becomes

(21) $$N_{\mathrm{combined}}=N_{\mathrm{binary}}+N_{\mathrm{multi-class}}=N_{\mathrm{binary}}\left(1+\gamma \right)$$

where $\gamma =\max(\bar{\alpha },\bar{\beta })+\delta$ accounts for pathologies requiring either subtype or severity classification, with δ representing an overlap adjustment for pathologies requiring both. Estimating γ∈[0.50,0.70] based on the observation that most pathologies requiring characterization need at least one of subtype or severity classification:

(22) $$N_{\mathrm{combined}}\approx \left(1.50-1.70\right)\times N_{\mathrm{binary}}$$

The combined architecture thus offers a modest reduction in total classifier count relative to the cascaded approach, at the cost of increased per-classifier complexity and more demanding training data requirements. Neither architectural choice fundamentally alters the conclusion that comprehensive multi-class diagnostic coverage remains practically unattainable.

### 8.7. Summary: The Expanded Combinatorial Challenge

The analyses in this section demonstrate that extending from binary detection to clinically useful characterization substantially expands the combinatorial challenge facing comprehensive medical AI coverage.

Three architectural approaches have been examined. The binary-only architecture addresses pathology detection alone, answering the question of whether a specific condition is present. This architecture requires the classifier counts estimated in Section 7, ranging from 4500 under conservative assumptions to 150,000 under comprehensive assumptions. The cascaded architecture with separate subtype and severity classifiers adds two additional classification layers invoked when binary detection is positive. This architecture approximately doubles the total classifier requirement, ranging from 7650 under conservative assumptions to 315,000 under comprehensive assumptions. The combined architecture with joint subtype-severity classifiers reduces the total classifier count relative to the cascaded approach but increases the complexity of each multi-class classifier and its training data requirements. This architecture yields requirements ranging from 6750 under conservative assumptions to 255,000 under comprehensive assumptions.

These estimates must be compared against the current state of FDA-cleared AI medical devices, which number approximately 700 to 900 as of 2025 [34,35], although recent taxonomies tracking these clearances document up to 1016 authorizations [66]. However, scoping reviews highlight critical reporting gaps in these cleared devices [67] and raise concerns regarding their real-world generalizability across diverse clinical settings [68]. The coverage ratio under any architecture and any estimation scenario falls below one percent, typically ranging from 0.2 to 0.7 percent of the classifiers required for comprehensive diagnostic coverage.

Table 14 presents this comparison across architectures and estimation scenarios, illustrating that the gap between current capabilities and comprehensive coverage spans two to three orders of magnitude regardless of architectural choices.

This analysis demonstrates that “clinically complete AI coverage through task-specific classifiers is not merely impractical but effectively unattainable.” Critically, as established in Section 2, general-purpose LLMs cannot substitute for this combinatorial space of validated classifiers: their demonstrated propensity for committing fundamental diagnostic errors [20], inability to agree on radiological ground truth, and lack of regulatory clearance for autonomous diagnostic use preclude their deployment as classifier surrogates. The theoretical elegance of LLM-to-classifier routing architectures (Figure 9) should not obscure the practical reality that comprehensive implementation would require classifier development efforts exceeding current capabilities by two to three orders of magnitude, i.e., a gap that cannot be bridged by substituting unvalidated general-purpose systems for validated task-specific tools. This finding reinforces the argument developed in the following Section 11 that AI development should prioritize predictive rather than classificatory applications, where population-level risk patterns more naturally align with the statistical foundation of machine learning systems.

### 8.8. Retrieval-Augmented Generation and Tool-Augmented Orchestration

**Figure 12:**
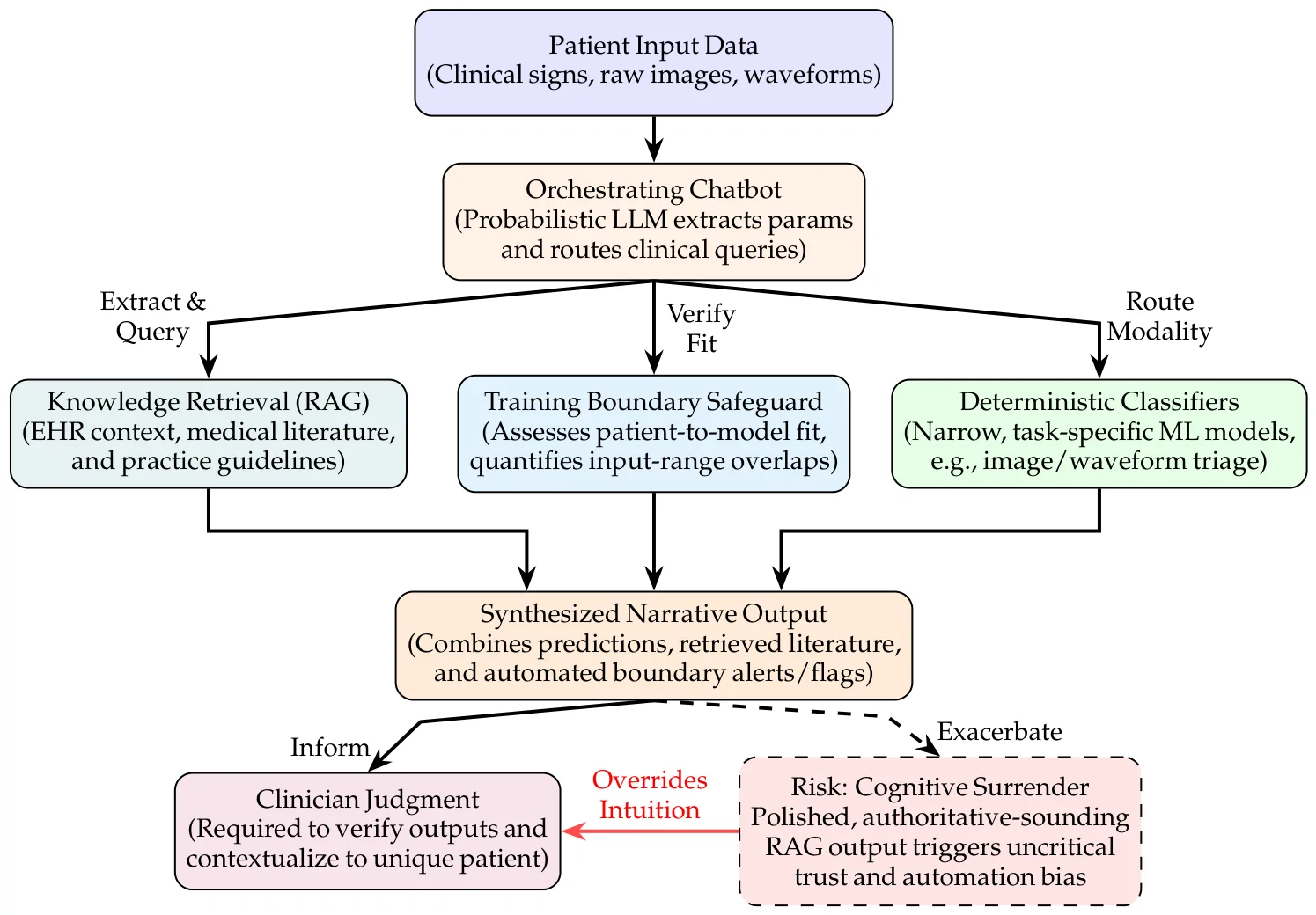
Workflow architecture of a Retrieval-Augmented Generation (RAG) chatbot and tool-augmented orchestration system. The general-purpose LLM acts as an orchestrating interface, routing medical queries, querying external knowledge bases, and executing task-specific classifiers. Crucially, a boundary safeguard checks how well the patient’s feature profile fits the classifier’s training distribution. The synthesized natural language report is delivered to the clinician. However, the high fluency of this output introduces a serious risk of cognitive surrender, where clinicians override their own judgment in favor of incorrect AI-generated conclusions [53,55].

The practical implementation of the theoretical routing workflows described above is increasingly operationalized through Retrieval-Augmented Generation (RAG) and tool-augmented conversational architectures, as illustrated in Figure 12. In these frameworks, a general-purpose language model functions not as an autonomous clinical diagnostic engine, but rather as an orchestrating user interface. When presented with an unstructured clinical query or a patient data stream, the model serves as a natural language processing layer that extracts key clinical parameters and coordinates downstream tools. Rather than generating a response based solely on its frozen training parameters, the chatbot retrieves contextually relevant information from verified medical databases, guidelines, or electronic health records to anchor the conversational output. In multimodal applications, this orchestrating chatbot acts as a gateway that routes specific raw data, such as chest radiographs or electrocardiograms, to task-specific deterministic classifiers. The orchestrator then receives the classifier output, synthesizes it with the retrieved clinical literature, and generates a cohesive, natural language report for the clinician.

While this tool-augmented RAG architecture represents a meaningful technical advancement in clinical workflow integration, it does not resolve the fundamental epistemological mismatch between population-level statistics and individual patient care. Crucially, like the simpler routing workflows, this complex orchestration architecture remains purely theoretical, and its comprehensive clinical realization is restricted by a massive “implementation gap” due to the absolute scarcity of validated downstream classifiers. The specialized classification models invoked by the conversational interface remain bound by their learned decision boundaries and the statistical distributions of their training cohorts. Consequently, before any algorithmic prediction is accepted as clinically actionable, each new specific patient must first be assessed to determine whether, and to what extent, their unique feature profile falls within the representation of the model’s training dataset. Without this initial screening of training distribution proximity, the clinical reliability of any downstream prediction remains entirely unknown.

More critically, the integration of statistical classifier predictions into a highly fluent, contextually rich, and authoritative-sounding conversational narrative may dangerously exacerbate the clinical safety risks detailed throughout this manuscript. By presenting diagnostic estimates alongside retrieved medical guidelines in a polished natural language format, RAG chatbots can easily induce cognitive surrender among clinical users, leading to the uncritical adoption of algorithmic outputs with minimal scrutiny [53]. Clinicians and trainees are highly susceptible to accepting these beautifully structured, seemingly comprehensive syntheses without independent verification, overriding their own clinical intuition and reasoning even when the AI-generated advice is misleading [50,51,55,56]. This uncritical acceptance risks overlooking the reality that the individual patient in front of them may represent an out-of-distribution exception to the statistical rule.

## 9. Critical Appraisal Framework for AI Tools

### 9.1. Essential Questions for AI Evaluation

Given the fundamental limitations of population-trained models applied to individual patients, clinicians must develop competency in critically appraising AI tools. The essential questions include:

**Q1.**  Training Data: On what population was this model trained? What were the inclusion/exclusion criteria? What demographic, geographic, and clinical characteristics were represented?
**Q2.**  Validation Methodology: How was the model validated? Was there external validation on independent populations? Was temporal validation performed?
**Q3.**  Performance Metrics: What are the reported sensitivity, specificity, positive/negative predictive values? At what prevalence were these calculated? How do they translate to my patient population?
**Q4.**  Failure Modes: What are the known failure modes? What types of cases does the model systematically misclassify? Are there documented biases?
**Q5.**  Generalizability: Is there evidence that the model performs consistently across demographic subgroups? Has it been validated in populations similar to the patient in front of me?
**Q6.**  Appropriate Use Cases: For what clinical scenarios was this tool designed? What are the boundaries of its intended use?

### 9.2. Warning Signs of Unreliable AI Systems

Beyond the structured evaluation framework outlined above, clinicians should develop pattern recognition for warning signs that suggest unreliable AI performance claims. These warning signs do not necessarily indicate that a system is without value, but they should prompt heightened scrutiny and requests for additional validation evidence before clinical adoption.

The most significant warning sign is the claim of perfect or near-perfect accuracy. Real-world clinical data contains inherent noise, ambiguous cases, and interobserver disagreement even among expert clinicians. Claims of accuracy exceeding 95 to 99 percent should trigger suspicion of data leakage (where information about the outcome inadvertently appears in the input features), overfitting to the training set, or evaluation on trivially separable cases that do not reflect the diagnostic challenges encountered in practice. When independent external validation is performed, accuracy typically decreases substantially from development-set performance.

Single-institution validation represents another significant concern. Models developed and validated at a single institution may learn institution-specific artifacts rather than generalizable disease patterns. These artifacts can include imaging equipment characteristics, documentation conventions, patient population demographics, and even the physical layout of clinical spaces reflected in imaging protocols. Without multi-site validation, the degree to which reported performance generalizes to other clinical environments remains unknown.

The absence of external validation entirely should prompt particular caution. Internal validation techniques such as cross-validation and bootstrap methods estimate performance on data drawn from the same distribution as training data. They cannot assess whether the model will perform adequately when deployed in environments with different patient populations, equipment, or clinical practices. External validation on independent datasets from different institutions and time periods provides the only reliable estimate of real-world generalizability.

Unreported subgroup performance may mask significant disparities in model behavior across demographic groups. A model with acceptable aggregate performance may systematically underperform for minority populations, older patients, patients with multiple comorbidities, or other subgroups underrepresented in training data. Responsible reporting includes stratified performance metrics across clinically and demographically relevant subgroups.

Finally, validation performed on data drawn from the same distribution as training data, even if technically held out, does not test the model’s ability to generalize to genuinely new patient populations. Temporal validation (testing on data collected after the training period) and geographic validation (testing on data from different institutions or regions) provide stronger evidence of generalizability than random holdout sets drawn from the same data collection effort. Table 15 summarizes these warning signs and their implications for clinical AI evaluation.

Established reporting guidelines provide structured frameworks for critical appraisal of AI tools. The TRIPOD+AI statement offers updated guidance for reporting clinical prediction models developed using regression or machine learning and is essential for transparent reporting, external validation, and prediction-model appraisal [69]. The PROBAST tool provides a framework for assessing risk of bias and applicability in prediction-model studies [70]. For clinical trials evaluating AI interventions, the CONSORT-AI reporting guideline extension supports trial standards that capture human–AI interaction and clinical workflow effects [71], complemented by the SPIRIT-AI extension for clinical trial protocols [72]. For early-stage clinical evaluation of AI decision-support systems, the DECIDE-AI reporting guideline is useful because many AI tools enter clinical environments before large trials [73]. The MI-CLAIM checklist provides minimum information needed to assess clinical AI models for training data, validation, performance metrics, and intended use [74]. The FUTURE-AI international consensus guideline for trustworthy clinical AI emphasizes fairness, universality, traceability, usability, robustness, and explainability across the AI lifecycle [75]. Methodological review has shown inconsistent use of the term “validation” in medical AI imaging studies, arguing that “validated” can mean very different things and should not be accepted without checking whether validation was external, temporal, or independent [76].

### 9.3. The Dangerous Graduate: A Risk Profile

This analysis identifies the “most dangerous graduate” profile: one who trusts AI implicitly, accepts outputs because they “sound authoritative,” and fails to recognize when AI recommendations require verification.

**Figure 13:**
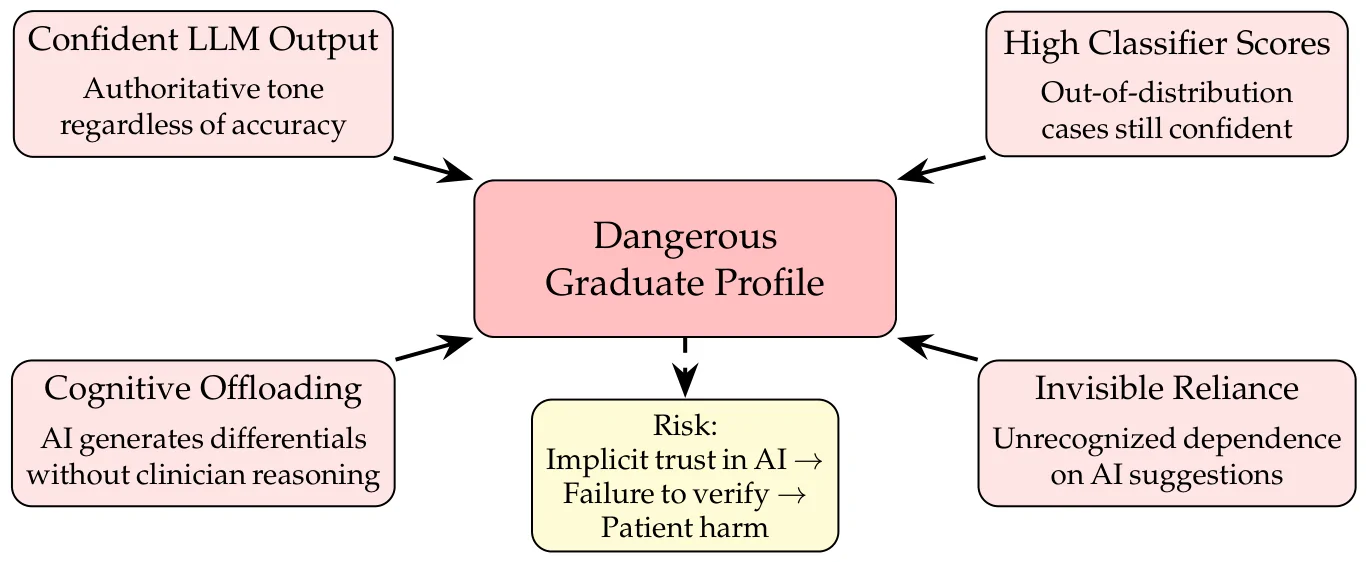
Four converging factors that create the “dangerous graduate” risk profile: authoritative-sounding LLM outputs, overconfident classifier scores on out-of-distribution cases, cognitive offloading of reasoning to AI, and invisible reliance on algorithmic suggestions.

This risk is amplified by four converging factors. LLM outputs sound authoritative and confident regardless of their accuracy, creating a false sense of reliability. Deterministic classifiers compound this problem by providing high confidence scores even when applied to out-of-distribution cases for which they were never validated. Cognitive offloading emerges when clinicians allow AI to generate differential diagnoses without engaging in the reasoning process themselves, atrophying the very skills that safe AI use requires [54]. Finally, invisible reliance develops when AI outputs become so integrated into workflow that clinicians no longer recognize their dependence on algorithmic suggestions. Figure 13 illustrates how these four factors converge to create the dangerous graduate risk profile.

### 9.4. Required Competencies for AI-Era Physicians

The preceding analysis of warning signs and the dangerous graduate profile establishes the need for explicit competency development in physician training and continuing education. Six core competency domains emerge as essential for effective physician collaboration with AI systems in clinical practice, as summarized in Table 16.

The foundational competency is AI literacy, which encompasses understanding the distinction between probabilistic systems (such as large language models) and deterministic task-specific classifiers, recognizing the shared limitations that arise from population-level training, and knowing the appropriate use cases for each system type. Without this foundational understanding, clinicians cannot make informed decisions about when to rely on AI outputs and when to exercise independent judgment.

Critical appraisal competency builds on AI literacy by enabling clinicians to evaluate specific AI tools rather than AI as an abstract category. This domain requires skills in assessing training data composition and representativeness, validation methodology and its limitations, reported performance metrics and their applicability to local patient populations, documented failure modes and systematic misclassification patterns, and the strength of generalizability claims. These skills parallel the critical appraisal competencies long required for evaluating clinical research, extended to the specific characteristics of algorithmic tools.

Integrative reasoning represents the cognitive skill of synthesizing AI outputs with the full clinical context available to the treating physician. This competency includes recognizing atypical presentations that may fall outside the training distribution of AI systems, managing the complexity introduced by competing comorbidities that may confound algorithmic predictions, and weighing AI suggestions against information unavailable to the algorithm. Integrative reasoning is the practical manifestation of the principle that AI informs but does not conclude.

Uncertainty tolerance addresses the psychological and professional capacity to operate effectively in conditions of ambiguity. Clinicians must develop comfort with probabilistic outputs that do not provide categorical certainty, willingness to acknowledge the limits of knowledge (including the limits of AI knowledge), and skill in deploying confirmatory testing appropriately when AI outputs require verification. This competency directly counters the dangerous graduate profile characterized by inappropriate certainty derived from authoritative-sounding AI outputs.

Human connection encompasses the interpersonal competencies that AI systems cannot replicate: communication skills that build trust and elicit accurate histories, the ability to explore goals of care and values that inform treatment decisions, and sensitivity to subtle cues from patient interaction that inform clinical reasoning. These competencies become more rather than less important as AI assumes greater roles in pattern recognition and data processing, because the distinctively human elements of care become the primary differentiator of physician contribution.

Metacognitive awareness represents the highest-order competency: the ability to monitor one’s own reasoning processes, recognize when AI may be unreliable for the specific case at hand, and make deliberate decisions about when to override algorithmic recommendations. This competency requires continuous self-assessment and intellectual humility, acknowledging that both human and artificial intelligence have characteristic failure modes that must be actively monitored.

## 10. The Diagnostic Workflow: Where AI Fits and Where It Cannot

The preceding sections have established the theoretical limitations of AI systems for diagnostic medicine. This section examines how these limitations manifest practically across the stages of the diagnostic workflow, identifying where AI can contribute value and where physician judgment remains essential.

The diagnostic workflow comprises seven interconnected stages, each with distinct information requirements, cognitive demands, and decision-making characteristics. Analysis of AI integration potential across these stages reveals a consistent pattern: AI can augment physician capabilities at each stage, but fundamental aspects of clinical care remain beyond current and foreseeable AI capabilities.

History taking represents the initial stage of diagnostic reasoning. AI contributions in this domain center on ambient documentation technologies that transcribe and structure patient–physician conversations, reducing documentation burden and allowing physicians to maintain focus on the patient interaction. However, the core functions of history taking extend beyond information recording. Physicians elicit subtle diagnostic cues through follow-up questions prompted by patient responses, build rapport that encourages disclosure of sensitive information, and gather nonverbal data through observation of patient affect, movement patterns, and physiological signs. These functions require human connection and real-time adaptive communication that current AI systems cannot replicate.

Differential diagnosis generation represents a domain where AI, particularly large language models, can contribute substantially. Given a constellation of symptoms and findings, AI systems can generate comprehensive differential diagnoses that may include conditions the physician might not immediately consider. However, the output of such systems represents pattern matching across training data rather than clinical reasoning. The differential serves as a starting point for physician evaluation, not a conclusion. Critical evaluation and contextualization of AI-generated differentials against the specific patient’s clinical context remains a physician responsibility.

Physical examination represents a domain with limited AI applicability given current technology. Although research explores AI analysis of recorded examination components (auscultation sounds, movement patterns, skin findings), the comprehensive physical examination involves tactile assessment, dynamic patient interaction, and integration with history that remains beyond AI capabilities. The physician’s ability to feel tissue texture, assess resistance, observe patient responses to examination maneuvers, and modify the examination based on findings constitutes an irreplaceable component of diagnostic assessment.

Test selection benefits from AI-based decision support algorithms that can identify indicated tests based on clinical presentations, flag redundant or low-yield testing, and ensure adherence to evidence-based guidelines. However, final ordering decisions require integration of patient-specific context including patient preferences, logistical constraints, cost considerations, and assessment of whether test results will actually change management. These integrative judgments involve value considerations and contextual factors that AI systems cannot fully capture.

Result interpretation represents perhaps the strongest current domain for AI contribution. Image pre-analysis can highlight abnormalities for radiologist attention. Laboratory value trending can identify clinically significant patterns. Structured reporting can ensure systematic evaluation of all relevant findings. However, the distinction between AI-provided data and clinical interpretation must be maintained. AI systems identify patterns in data; interpretation requires integration with clinical context, assessment of pre-test probability, and judgment about clinical significance. A statistically abnormal finding may be clinically insignificant in one patient and critically important in another. This contextual judgment defines interpretation.

Diagnosis represents the culmination of the diagnostic workflow. AI contributions include risk stratification models that estimate disease probability and differential ranking algorithms that order candidate diagnoses by likelihood. However, the final diagnostic determination integrates all preceding workflow stages with physician judgment about the specific patient. As established throughout this paper, population statistics provide probability estimates rather than individual diagnoses. The physician must determine whether the patient conforms to the statistical patterns or represents an exception requiring different diagnostic consideration.

Treatment planning extends beyond diagnosis to therapeutic decision-making. AI contributions include protocol suggestions based on diagnosis, drug interaction checking, dosing calculations, and identification of relevant clinical trial opportunities. However, final treatment decisions require discussion of goals of care with patients and families, shared decision-making that incorporates patient values and preferences, and judgment about therapeutic tradeoffs that involve considerations beyond algorithmic optimization.

Table 17 summarizes AI contributions, remaining physician roles, and the reasons AI cannot replace physician judgment at each workflow stage.

The evidence synthesis across these workflow stages supports a consistent conclusion: physician–machine collaboration outperforms either alone, but only when the collaboration is structured appropriately. Studies demonstrate that physicians using AI as one input among many, while maintaining critical evaluation of AI outputs, outperform both physicians who ignore AI entirely and physicians who defer to AI without independent assessment. This finding reinforces the central argument that AI should inform and guide clinical reasoning without replacing the judgment that integrates AI outputs with the full clinical context.

## 11. The Predictive Paradigm: Aligning AI Capabilities with Clinical Utility

The combinatorial analyses in Section 7 and Section 8 demonstrate that comprehensive diagnostic coverage through task-specific classifiers is practically unattainable. More fundamentally, even if such coverage were achieved, the core limitation remains: “population-trained classifiers cannot be reliably applied to specific patients for diagnostic conclusions.” This finding suggests a fundamental reorientation: rather than pursuing the computationally and logistically infeasible goal of building classifiers for every (o,m,p) combination, AI development in medicine should prioritize “predictive” over “classificatory” applications.

### 11.1. Classification Versus Prediction: A Critical Distinction

Classificatory applications attempt to answer: “What pathology does this patient have?” This question requires validated classifiers for each organ-modality-pathology combination, yielding the combinatorial explosion documented above. In contrast, predictive applications answer questions such as

- “What is this patient’s risk of developing condition *X* within timeframe *T*?”
- “Given diagnosis *D*, what is the probability of treatment response to intervention *I*?”
- “What is this patient’s prognosis based on current clinical trajectory?”

The distinction is epistemologically significant:

- Classification (Diagnostic): “Does this patient have pneumonia?” → Requires high confidence for individual decision-making; false positives and negatives have immediate clinical consequences; confirmatory testing remains critical.
- Prediction (Prognostic): “Is this patient on a trajectory toward developing heart failure?” → Operates at the level of risk stratification; tolerates probabilistic uncertainty; triggers enhanced surveillance rather than immediate intervention.

To be clear, both paradigms can yield probabilistic outputs, and we do not claim otherwise. The epistemological advantage we ascribe to prediction is not that classification is “less statistical,” but that the clinical actions triggered by each paradigm differ fundamentally in their stakes and reversibility. When a classifier’s categorical output informs an immediate treatment decision, errors carry direct clinical consequences. When a predictor’s probabilistic output triggers enhanced monitoring, errors represent missed prevention opportunities, which is still consequential, but typically recoverable through the surveillance process itself. This asymmetry in error consequences, rather than any difference in statistical foundations, constitutes prediction’s pragmatic superiority for AI deployment.

### 11.2. The Epistemological Advantage of Prediction

**Figure 14:**
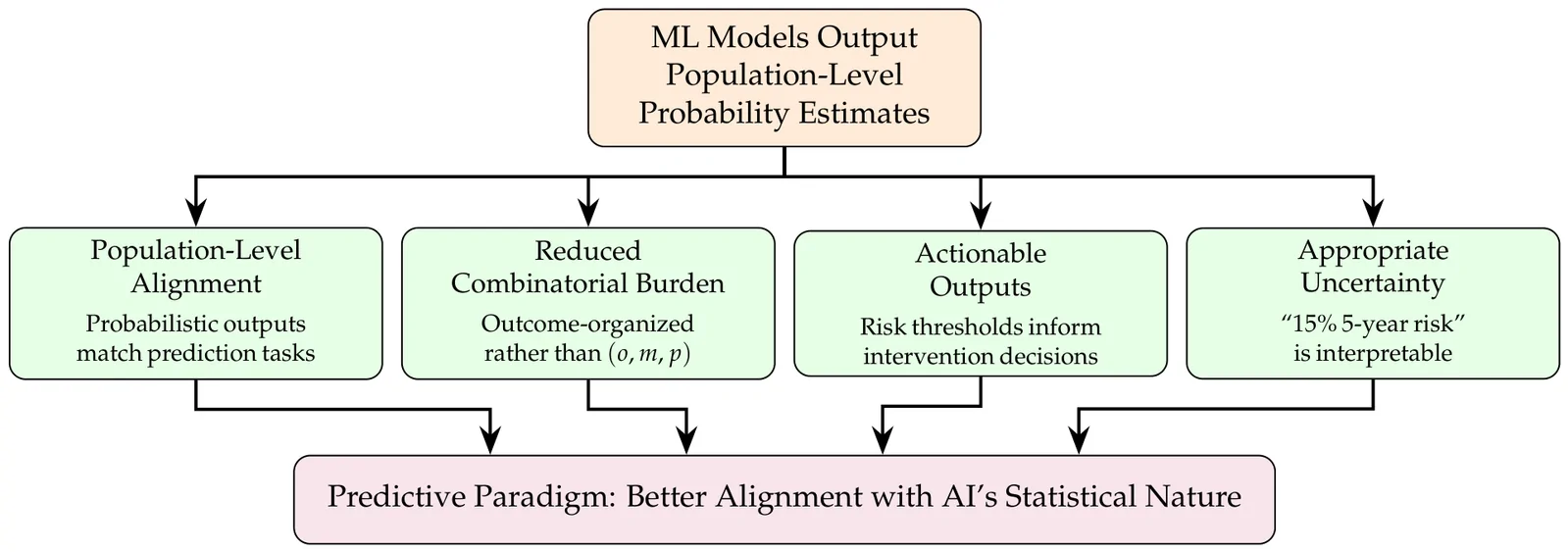
Four epistemological advantages of predictive applications over classificatory applications in medical AI: population-level alignment, reduced combinatorial burden, actionable outputs, and appropriate uncertainty representation; all flowing from the fundamental match between probabilistic AI outputs and predictive clinical questions.

To reiterate, both classificatory and predictive paradigms are statistical in nature and can produce probabilistic outputs; the distinction is not that one is “more statistical” than the other. Rather, the critical difference lies in how outputs are clinically interpreted and the consequences of errors. Predictive questions are better aligned with the clinical deployment of machine learning for several interconnected reasons:

Predictive questions are fundamentally better suited to the statistical foundations of machine learning for several interconnected reasons. First, machine learning models trained on population data inherently produce population-level probability estimates; prediction tasks explicitly require such probabilistic outputs, whereas classification tasks demand categorical decisions that population statistics cannot directly provide. Second, predictive models can be organized around clinical outcomes (e.g., cardiovascular events, cancer recurrence, treatment response) rather than requiring the full combinatorial cross-product of organs, modalities, and pathologies that diagnostic classification demands. Third, risk predictions directly inform clinical decision-making by identifying prophylactic intervention thresholds without requiring the categorical certainty that diagnosis demands. Fourth, predictive outputs naturally express uncertainty in clinically interpretable terms (e.g., “15% five-year mortality risk”), whereas diagnostic outputs often obscure the probabilistic nature of the underlying inference behind categorical labels. Figure 14 illustrates how these four advantages flow from the fundamental alignment between probabilistic AI outputs and predictive clinical questions.

**Figure 15:**
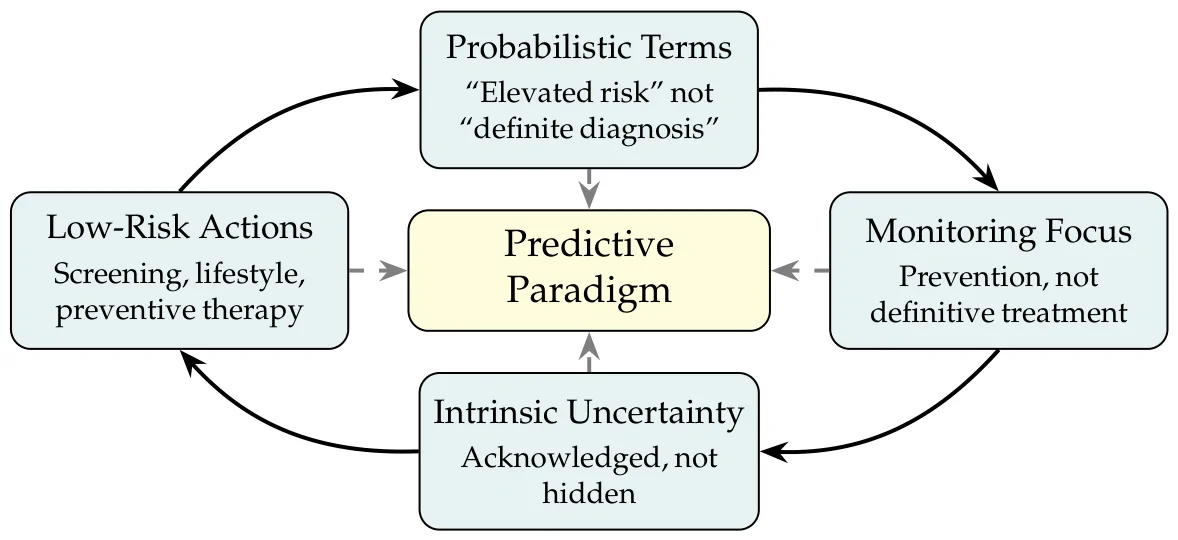
The predictive paradigm creates alignment between AI capabilities and clinical utility through four reinforcing mechanisms: probabilistic framing, monitoring-focused outputs, acknowledgment of intrinsic uncertainty, and low-risk intervention triggers.

Additionally, predictive applications align more naturally with the population-statistical nature of AI systems through several complementary mechanisms. Predictions inherently operate in probabilistic terms, communicating “elevated risk” rather than “definite diagnosis,” which matches the statistical outputs that machine learning systems actually produce. Predictive outputs trigger monitoring and prevention protocols rather than definitive treatment decisions, reducing the stakes of individual predictions while maintaining clinical utility. Furthermore, the predictive paradigm acknowledges uncertainty as intrinsic to forecasting rather than treating it as a limitation to overcome, thereby setting appropriate expectations for AI performance. Finally, predictive interventions are typically low-risk (e.g., enhanced screening protocols, lifestyle modification counseling, preventive therapies) meaning that even imperfect predictions yield net clinical benefit without the catastrophic consequences that misdiagnosis can entail. Figure 15 illustrates how predictive framing creates a virtuous cycle of appropriate AI deployment.

Predictive deep learning studies using electronic health records for clinical outcomes such as mortality and readmission provide useful examples of AI’s potential strength in prognostic risk prediction rather than narrow binary diagnosis [77]. Prognostic AI studies for continuous prediction of future acute kidney injury from electronic health-record data represent strong examples of clinically useful predictive AI that identifies adverse trajectories before obvious clinical deterioration [78]. Prognostic deep learning predicting pancreatic cancer risk from longitudinal disease trajectories supports the argument that AI may be especially valuable for early risk identification and surveillance [79]. Methodological work distinguishing diagnosis from prognosis in clinical prediction models directly supports the argument that predictive/prognostic AI is conceptually different from diagnostic classification [80].

### 11.3. Comparative Framework

The distinction between classificatory and predictive paradigms can be systematically analyzed across multiple dimensions that together define how AI systems interact with clinical practice.

The core question addressed by each paradigm differs fundamentally. Classification asks whether disease X is present now, requiring a determination about current pathological state. Prediction asks whether disease X will develop, requiring a forecast about future trajectory. This temporal distinction has profound implications for how AI outputs should be interpreted and acted upon.

The output interpretation requirements differ correspondingly. Classification demands binary or categorical determination: the patient either has the condition or does not, possibly with severity grading. Prediction produces probabilistic risk estimates: the patient has a certain probability of developing the condition within a specified timeframe. The categorical nature of classification creates pressure for high-confidence outputs, while the probabilistic nature of prediction explicitly acknowledges uncertainty.

The clinical actions triggered by each paradigm differ in their stakes and reversibility. Classification outputs inform definitive treatment decisions: initiating chemotherapy, performing surgery, prescribing disease-specific medications. Prediction outputs trigger enhanced surveillance and prevention: more frequent screening, lifestyle modification counseling, prophylactic interventions. The consequences of classification errors are typically more immediate and severe than the consequences of prediction errors, which generally represent missed prevention opportunities rather than direct iatrogenic harm.

Tolerance for uncertainty differs substantially between paradigms. Classification, because it informs definitive treatment, requires confirmation before irreversible interventions. Clinicians appropriately demand high confidence before acting on classificatory AI outputs. Prediction, because uncertainty is inherent to forecasting, operates with acknowledged probabilistic bounds. A 30 percent five-year risk estimate is clinically useful even though it explicitly acknowledges 70 percent probability that the adverse outcome will not occur.

Alignment with AI’s statistical nature differs between paradigms. Classification aligns poorly with the population-trained, probability-outputting nature of machine learning systems. Patients require categorical diagnoses; AI provides probability estimates. Prediction aligns well with AI’s statistical foundation. Patients and clinicians can act on risk estimates directly; AI provides exactly such estimates.

Table 18 summarizes this comparative analysis across all key dimensions.

To illustrate how the predictive paradigm reframes AI applications across clinical domains, Table 19 presents paired classificatory and predictive formulations for common clinical scenarios. In each case, the predictive formulation asks a question that is both better aligned with AI capabilities and often more clinically actionable than the classificatory formulation. Knowing that a patient has a 15 percent five-year stroke risk given atrial fibrillation burden may be more useful for anticoagulation decisions than a binary determination of whether atrial fibrillation is present on a single ECG. Knowing the probability of treatment response to specific therapeutic options may be more useful for oncological decision-making than a binary determination of malignancy presence.

### 11.4. Reframing AI Development Priorities

Rather than pursuing comprehensive classification coverage, which is both practically infeasible and epistemologically problematic, we propose that artificial intelligence development should prioritize predictive power over diagnostic classification accuracy. Under this reframed paradigm, the strategic goal of clinical technology shifts from detecting present pathology to warning clinical teams when a patient is on an adverse trajectory toward developing a given pathology. This conceptual reorientation acknowledges that artificial intelligence systems trained on population-wide data are fundamentally better suited to identifying statistical risk patterns across populations than making definitive, individual diagnostic determinations.

**Figure 16:**
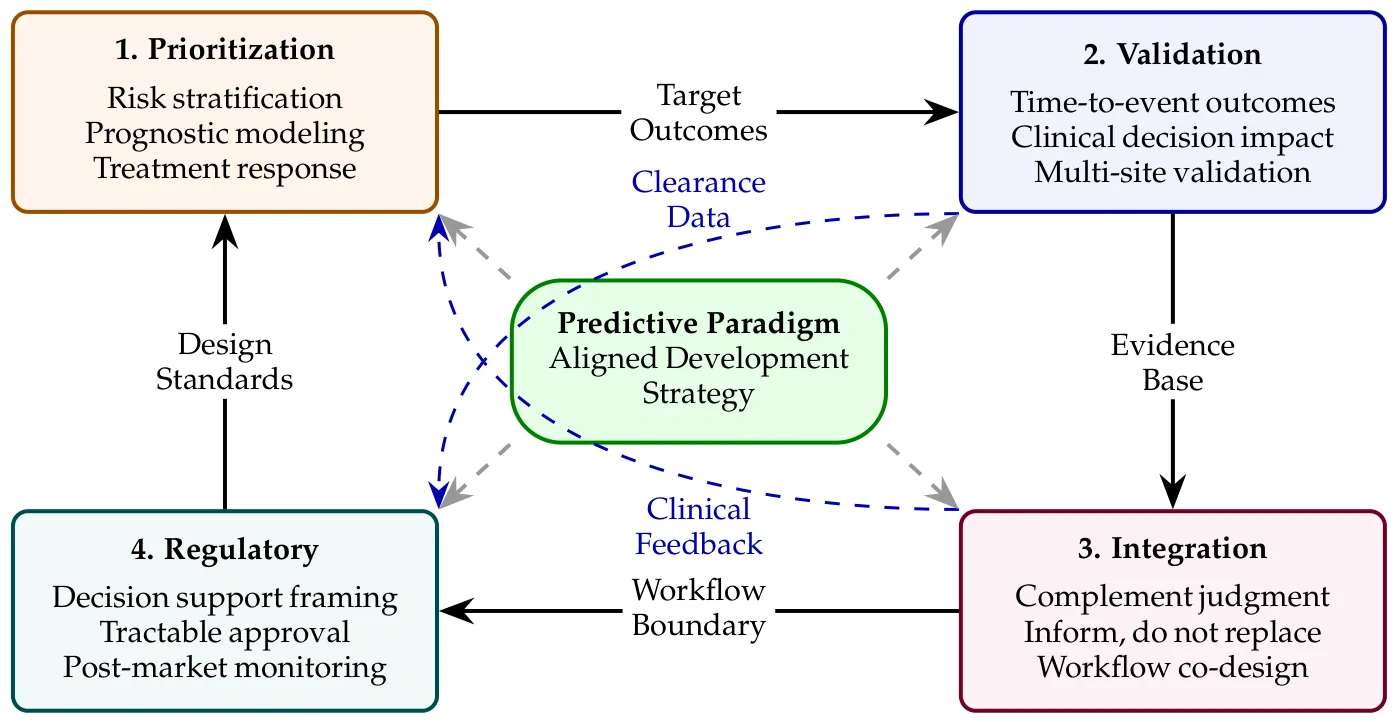
Operational and relational flowchart of the predictive paradigm for medical AI development. Rather than functioning as isolated steps, the four domains form a continuous, feedback-driven lifecycle. Strategic prioritization of prognostic tools generates clear targets for time-to-event validation, which establishes the clinical evidence base required for safe workflow integration. In turn, clinical integration boundaries dictate the appropriate regulatory approval pathway. Cross-relational links show how validation data directly feeds into regulatory clearance, and how real-world clinical integration requirements guide the prioritization of future algorithm development.

The predictive paradigm has significant implications for resource allocation in medical AI development across four interconnected domains. With respect to “prioritization”, development efforts should emphasize risk stratification, prognostic modeling, and treatment response prediction over autonomous diagnostic classification; redirecting the field toward applications where AI’s statistical nature provides genuine clinical value. Regarding “validation endpoints”, predictive models should be validated against time-to-event outcomes and clinical decision impact rather than diagnostic accuracy alone, ensuring that performance metrics reflect real-world utility. The “integration strategy” implications are equally significant: predictive AI naturally complements physician decision-making by providing probability estimates that inform but do not replace clinical judgment, whereas classificatory AI implicitly competes with physician expertise. Finally, from a “regulatory pathway” perspective, predictive tools that inform rather than diagnose may face more tractable regulatory pathways because they function as decision support rather than autonomous diagnostic devices. Figure 16 illustrates how these four considerations reshape the medical AI development landscape.

Clinical prediction model evaluation covering model development, internal validation, internal–external validation, and external validation is useful for explaining why generalizability and transportability must be tested before applying population-trained models to new patients [81]. Guidance on how to undertake external validation studies of clinical prediction models supports the need to test model transportability, calibration, discrimination, and clinical usefulness before applying prediction models to new patients or populations [82]. Decision curve analysis, which evaluates whether prediction models improve clinical decision-making through net benefit, strengthens the predictive-paradigm argument by distinguishing clinical usefulness from accuracy or AUC alone [83].

This paradigm shift does not diminish the value of task-specific diagnostic classifiers where they exist and are validated; rather, it argues against pursuing comprehensive diagnostic coverage as the primary goal of medical AI development. The approximately 850 FDA-cleared AI devices [34,35] represent valuable tools within their validated scope, but the combinatorial gap documented in this analysis suggests that closing this gap through classifier proliferation is neither feasible nor necessarily desirable. The predictive paradigm offers an alternative path forward that better aligns the statistical nature of machine learning with the probabilistic realities of clinical medicine.

## 12. Discussion

### 12.1. Implications for Equity and Access

AI in medicine presents dual potential for health equity: amplifying or reducing disparities. Landmark bias studies of commercial population-health algorithms have shown racial bias because healthcare cost was used as a proxy for health need, providing a powerful example of how models trained on population-level proxy outcomes can harm individual and subgroup care [84]. Bias studies showing that gender imbalance in medical imaging datasets can produce biased classifiers support the section on demographic imbalance, dataset composition, and subgroup performance [85]. Perspectives outlining sources of algorithmic bias and fairness challenges in medical AI, including data acquisition, genetic variation, labeling variability, and clinical workflow inequities, provide a broad fairness framework [86].

The disparity-amplifying risks are substantial. Models trained predominantly on data from well-resourced health systems may perform poorly when deployed for underrepresented populations whose clinical presentations, disease prevalences, and healthcare access patterns differ from training distributions. Sophisticated AI tools may concentrate in wealthy institutions with the infrastructure, technical expertise, and capital to implement them, thereby creating two-tiered care that advantages already-privileged populations. Algorithmic bias may systematically disadvantage minority patients through training data that reflects historical inequities, proxy variables that encode protected characteristics, and validation studies that fail to assess subgroup performance. Most fundamentally, the population-statistics problem documented throughout this paper disproportionately affects patients whose presentations fall outside the “typical” patterns on which models were trained, i.e., precisely the patients who already face barriers to equitable care.

**Figure 17:**
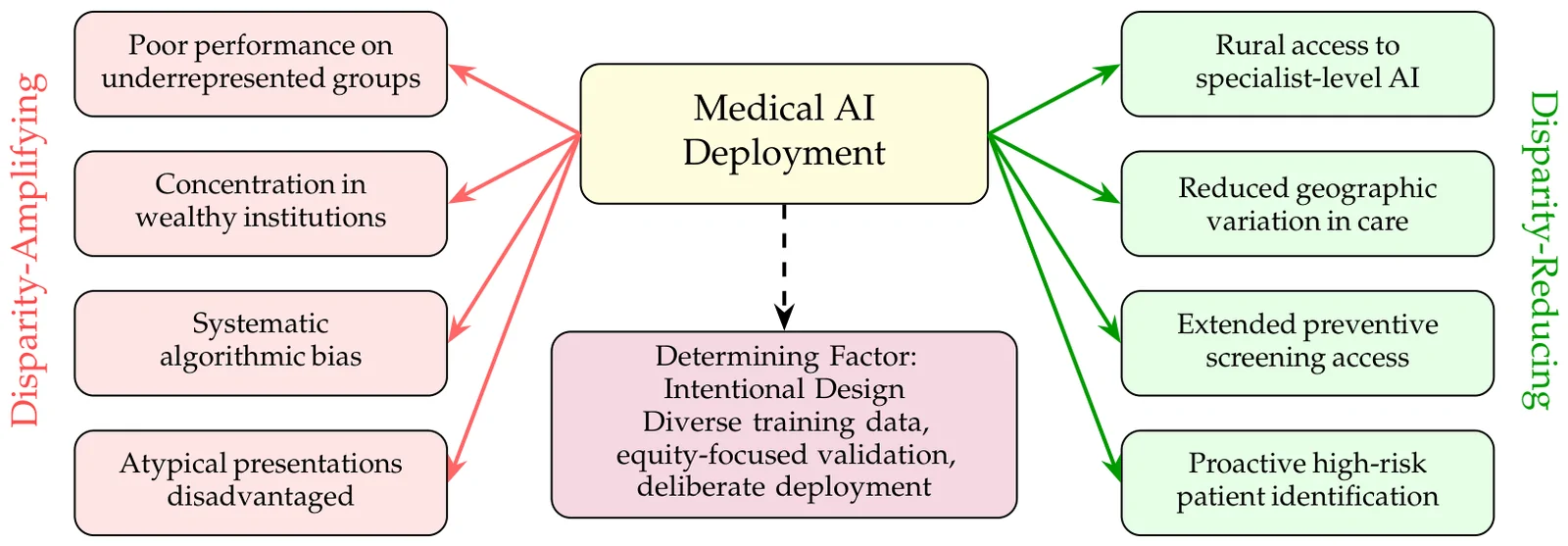
The dual potential of medical AI for health equity: disparity-amplifying risks (**left**)versus disparity-reducing opportunities (**right**), both stemming from medical AI deployment (**center**). The determining factor (**bottom**) is intentionality in development and deployment; without deliberate equity focus, AI will likely amplify existing disparities.

Conversely, AI presents genuine disparity-reducing opportunities if deployed with intentionality. Rural and under-resourced settings that lack specialist expertise could access specialist-level pattern recognition through AI tools, potentially democratizing diagnostic capabilities. Standardized decision support could reduce the geographic variation in care quality that currently disadvantages patients in under-resourced regions. AI-assisted screening programs could extend preventive care access to populations who currently lack it, identifying disease at earlier and more treatable stages. Predictive tools could identify high-risk patients for proactive intervention before they present with advanced disease, potentially addressing disparities that arise from delayed care-seeking. Figure 17 illustrates this dual potential and the determining role of intentional design.

The determining factor is “intentionality in development and deployment”. The STANDING Together consensus recommendations for transparency and diversity in health datasets used for AI strengthen the equity section by supporting dataset documentation, representativeness, and subgroup evaluation [87]. Frameworks describing digital determinants of health and how digital health tools can widen disparities if access, infrastructure, and equity are not built into design further support the claim that AI may either reduce or amplify disparities depending on intentional design choices [88]. Without rigorous validation across diverse populations and deliberate equity focus, AI will likely amplify disparities.

### 12.2. Synthesis: The Irreducible Role of Clinical Judgment

The analyses presented in this paper establish several key findings regarding AI in clinical medicine that collectively define the boundaries of appropriate AI integration. The foundational finding is that both probabilistic and deterministic AI systems are trained on population data but deployed for individual patient care, creating an irreducible epistemological gap that no technical advancement can fully close. Building on this foundation, the combinatorial analyses demonstrate that comprehensive binary classification coverage is practically unattainable, requiring an estimated 50,000–150,000+ distinct validated classifiers (Table 9 and Table 13), i.e., orders of magnitude beyond current development. Yet even with comprehensive coverage, the fundamental limitation would persist: population statistics cannot determine whether a specific patient conforms to learned patterns.

**Figure 18:**
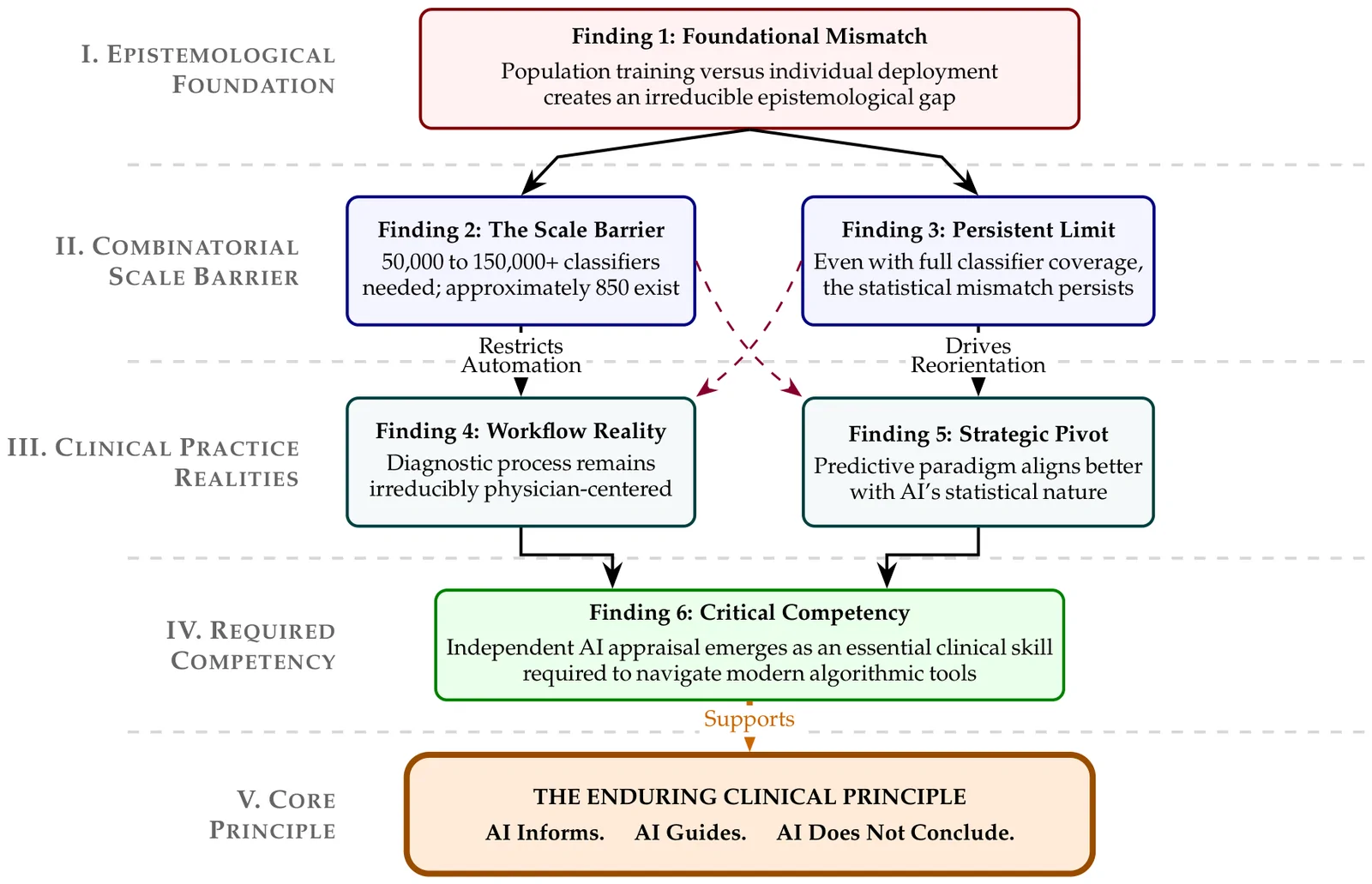
Synthesis of the manuscript’s six key findings. The foundational mismatch (Finding 1) establishes the theoretical scale barrier (Finding 2) and persistent statistical limits (Finding 3) of clinical classifiers. These technical constraints translate into practice as a physician-centered workflow (Finding 4) and dictate a strategic shift to a predictive paradigm (Finding 5). Crucially, the cross-relational links demonstrate that scale limitations directly motivate the predictive pivot, while statistical uncertainty necessitates human-in-the-loop workflows. Together, these factors require independent critical appraisal competency (Finding 6), ultimately culminating in the unshakeable clinical principle that AI informs and guides but does not conclude.

These theoretical limitations manifest practically in the finding that the diagnostic workflow remains physician-centered (Table 17): AI augments but cannot replace history-taking, clinical reasoning, confirmatory testing, and final diagnostic judgment. The constructive response to these limitations is the finding that predictive applications align better with AI capabilities than classificatory applications (Table 18), suggesting strategic reorientation toward identifying adverse trajectories rather than diagnosing present pathology. Finally, navigating this landscape requires that critical appraisal of AI outputs become an essential clinical competency (Table 16); that includes understanding training data, validation methodology, known limitations, and appropriate use cases for each tool. Figure 18 illustrates how these six findings form a coherent framework for understanding AI’s role in clinical medicine.

### 12.3. The Enduring Principle

The central conclusion of this paper can be stated simply:

AI informs. AI guides. AI does not conclude.

The core diagnostic act (i.e., integrating AI outputs with clinical context, ordering confirmatory testing, exercising judgment for the specific patient) remains irreducibly human. Both probabilistic and deterministic systems suffer from the same fundamental limitation: they are trained on population-wide data, but you are treating an individual, and you can never predict with certainty whether the patient in front of you fits the statistics.

The appropriate response is neither uncritical adoption nor reflexive rejection, but “thoughtful integration”: leveraging AI where it adds value while maintaining the clinical judgment, confirmatory testing, and human connection that define excellent patient care.

## 13. Conclusions

This position paper establishes that medical artificial intelligence systems are fundamentally bound by an irreducible population-to-individual epistemological gap, which prevents population-trained aggregate statistical models from prescribing definitive individual bedside decisions. By introducing a multi-dimensional combinatorial model, we demonstrate that achieving complete diagnostic automation under subspecialty-level clinical granularity would require an unfeasible 50,000–150,000+ distinct, task-specific classifiers, far exceeding current FDA clearances by orders of magnitude. Consequently, we argue for a strategic paradigm shift from diagnostic classification to predictive prognostic modeling, aligning the statistical nature of machine learning with clinical utility while reinforcing clinical judgment as the irreplaceable integrative agent in individual patient care. To facilitate the practical translation of these findings, a structured set of actionable, multi-stakeholder guidelines has been compiled in Appendix A.

While this framework offers a robust taxonomy distinguishing probabilistic and deterministic systems, its boundaries may blur as multimodal foundational models continue to develop and integrate clinical tasks; nevertheless, the mathematical limits of applying population statistics to unique individual patient profiles remain a persistent boundary. Furthermore, our quantitative estimates are modeled on current clinical practice divisions, which may evolve as digitized, collaborative clinical workflows are co-designed. Finally, future work must focus on the empirical characterization of AI performance on low-prevalence, rare conditions, the development of clinical interfaces that actively mitigate cognitive surrender, and the longitudinal integration of AI critical appraisal competencies within medical training curricula.

## Figures and Tables

**Table 1:** Characteristics of Probabilistic AI Systems in Clinical Applications.

Characteristic	Description
Operational Mechanism	Statistical pattern matching across training corpus
Output Nature	Probability distributions over possible responses
Training Data	Population-wide text including medical literature, clinical notes, educational materials
Clinical Analog	Initial hypothesis generation; differential diagnosis formulation
Fundamental Limitation	Outputs reflect aggregate population statistics, not individual patient-specific reasoning
Appropriate Use	Generating differentials as ‘starting points’ requiring verification

**Table 2:** Characteristics of Deterministic Classification Systems in Clinical Applications.

Characteristic	Description
Operational Mechanism	Supervised learning of decision boundaries from labeled data
Output Nature	Discrete categorical predictions (often with confidence scores)
Training Data	Curated datasets with expert-labeled ground truth annotations
Clinical Analog	Specific diagnostic tests with defined sensitivity/specificity
Fundamental Limitation	Performance reflects training population; may not generalize to individual patient
Appropriate Use	Pre-screening, risk stratification, augmenting (not replacing) clinical judgment

**Table 3:** Comparison of general-purpose multimodal LLMs versus task-specific machine learning models for medical image interpretation.

Characteristic	General-Purpose LLMs	Task-Specific ML Models
Training Paradigm	Massive, heterogeneous corpora spanning diverse domains; medical imaging content constitutes a small, potentially unrepresentative fraction	Curated datasets comprising thousands to millions of expert-annotated examples for a specific imaging modality and defined pathological conditions
Feature Optimization	Visual representations not optimized for specific diagnostic features; broad but shallow capabilities across many domains	Learned representations directly optimized for discriminative features relevant to the diagnostic task (e.g., hypodensity vs. hyperdensity, sulcal effacement vs. prominence)
Performance Characteristics	Largely unknown and unvalidated for radiological interpretation; propensity for fundamental diagnostic errors in specific case studies [20]; failure modes poorly characterized	Quantified sensitivity, specificity, and predictive values; validated against histopathological or clinically confirmed diagnoses; systematic failure mode analysis
Regulatory Status	Not validated or cleared for diagnostic imaging interpretation; no FDA authorization for autonomous clinical use	Multiple systems FDA-cleared (510(k) or De Novo) for specific indications; defined performance benchmarks and intended use populations [34,35]
Interpretability	Text-based explanations generated post-hoc; may not reflect actual visual features driving interpretation	Direct visual attention mapping (CAM, Grad-CAM) highlighting image regions driving predictions; enables verification of reasoning alignment [36]
Clinical Integration	No established workflows; outputs lack standardization for PACS integration; variable disclaimer inclusion	Purpose-built for clinical workflow integration; standardized outputs formatted for radiologist review and PACS compatibility [37]
Appropriate Applications	Report summarization, clinical documentation assistance, natural language interfaces, literature synthesis, workflow orchestration	Diagnostic triage, lesion detection and localization, quantitative analysis, second-reader applications within validated scope

**Table 4:** AI Integration Points in the Diagnostic Workflow.

Workflow Stage	AI Application	Role and Limitation
Differential Generation	LLM-based chatbots	Mirrors initial reasoning through probabilistic pattern-matching; starting point, not conclusion
Risk Stratification	Predictive algorithms	Surfaces patients needing attention; requires clinical verification
Image Analysis	Deterministic classifiers	Pre-analysis before radiologist review; produces false positives/negatives requiring expert interpretation
Documentation	Ambient AI scribes	Drafts documentation from clinical encounters; requires physician review and attestation
Decision Support	Integrated EHR algorithms	Alerts and recommendations; must be contextualized to specific patient

**Table 5:** Information Channels in Clinical Diagnosis: Physician Access versus Text-Based AI Access.

Information Channel	Physician	Text-Based LLM *
Written clinical notes	✓	✓
Visual observation of patient	✓	—
Facial expressions and affect	✓	—
Gait, posture, and movement	✓	—
Skin color, diaphoresis, cyanosis	✓	—
Auditory cues (voice, breathing)	✓	—
Physical examination findings	✓	—
Tactile assessment	✓	—
Olfactory cues	✓	—
Real-time patient interaction	✓	—
Nonverbal communication	✓	—
Clinical intuition from experience	✓	—

✓ = accessible; — = inaccessible; * While some observations could theoretically be documented in clinical notes, in practice such details are rarely recorded with sufficient specificity to support diagnostic reasoning.

**Table 6:** Requirements for Methodologically Valid AI-versus-Physician Diagnostic Comparison Studies.

Requirement	Rationale
Equivalent information access	Both AI and physician must receive the same information channels; if AI receives only text, physician performance on text-only tasks should be explicitly labeled as such
Ecological validity	Experimental conditions should approximate real clinical practice; if they do not, this limitation must be prominently disclosed
Appropriate framing of conclusions	Studies testing text interpretation should not generalize to claims about clinical diagnostic ability
Acknowledgment of modality limitations	Publications should explicitly state what information the AI system cannot access and how this affects generalizability
External validation	Findings should be replicated across diverse clinical settings before informing policy

**Table 7:** Representative Pathology Counts by Anatomical Region.

Anatomical	Estimated	Disease
Region	Pathologies	Examples
Lungs	40–60	Pneumonia, lung cancer (subtypes), COPD, fibrosis, TB, PE
Brain	50–80	Stroke, tumors, dementia (subtypes), MS, hemorrhage
Heart	40–60	Arrhythmias, cardiomyopathies, valve diseases, CAD
Liver	30–50	Hepatitis, cirrhosis, hepatocellular carcinoma, steatosis
Kidney	30–45	Glomerulonephritis, nephrolithiasis, RCC, PKD

**Table 8:** Diagnostic Modality Categories for AI Applications.

Category	Specific Modalities
Medical Imaging	Radiography (X-ray), CT, MRI, ultrasound, PET, SPECT, mammography
Ophthalmic Imaging	Fundoscopy, OCT, slit-lamp photography
Waveform Analysis	ECG, EEG, EMG, evoked potentials
Laboratory Values	Blood chemistry, hematology, biomarkers, genetic/genomic testing
Pathology	Histopathology, cytology, immunohistochemistry
Endoscopy	Upper/lower GI, bronchoscopy, cystoscopy
Other	Dermoscopy, capnography, pulse oximetry patterns

**Table 9:** Estimated Total Binary Classifiers Under Different Scenarios.

Scenario	|O|	Avg $|P_{o}|$	Avg Modalities	$N_{\mathrm{total}}$
Conservative	50	30	3	4500
Moderate	75	50	5	18,750
Comprehensive	100	75	8	60,000
Exhaustive (ICD-10 level)	120	100	10	120,000+

**Table 10:** Sensitivity Analysis: Effect of Dimensional Parameter Variation on Primary Estimate.

Parameter Varied	Low	Base	High	Estimate Range
Anatomical regions (|O|)	50	100	120	30,000–90,000
Pathologies per region ($|P_{o}|$)	30	75	100	24,000–80,000
Modalities per pair ($|M_{o,p}|$)	3	8	10	22,500–75,000
Combined multiplicative factor	1.5×	2.5×	3.5×	90,000–210,000
Primary range (all factors)	50,000–150,000+			

Base values: |O|=100, $|P_{o}|=75$, $|M_{o,p}|=8$, multiplicative factor =2.5×. Each row varies one parameter while holding others at base values.

**Table 11:** Representative Subtype Classification Requirements.

Organ	Pathology	Subtypes	$|T_{o,p}|$
Lung	Carcinoma	Adenocarcinoma, SCC, Small cell, Large cell, Carcinoid	5+
Breast	Carcinoma	Ductal, Lobular, Inflammatory, Paget’s, Phyllodes	5+
Brain	Glioma	Astrocytoma, Oligodendroglioma, Ependymoma, GBM	4+
Kidney	RCC	Clear cell, Papillary, Chromophobe, Collecting duct	4+
Lymphoma	—	Hodgkin, DLBCL, Follicular, Mantle cell, Burkitt	5+
Leukemia	—	AML, ALL, CML, CLL, Hairy cell	5+

**Table 12:** Representative Severity/Staging Classification Requirements.

Pathology Category	Staging System	Grades/Stages	$|S_{o,p}|$
Solid tumors (general)	TNM staging	T0–T4, N0–N3, M0–M1	5×4×2=40 *
Prostate carcinoma	Gleason grading	Grade Groups 1–5	5
Breast carcinoma	Nottingham grade	Grades I–III	3
Heart failure	NYHA classification	Classes I–IV	4
Chronic kidney disease	CKD staging	Stages 1–5	5
Liver cirrhosis	Child-Pugh score	Classes A, B, C	3
COPD	GOLD staging	Stages 1–4	4
Diabetic retinopathy	ETDRS scale	Levels 10–85 (simplified: 5)	5

* Often consolidated into stage groupings (I–IV) for practical classification.

**Table 13:** Estimated Total Classifiers with Cascaded Multi-class Architecture.

Scenario	$N_{\mathrm{binary}}$	$\bar{\alpha }$	$\bar{\beta }$	Multiplier	$N_{\mathrm{cascaded}}$
Conservative	4500	0.30	0.40	1.70	7650
Moderate	18,750	0.40	0.50	1.90	35,625
Comprehensive	60,000	0.45	0.55	2.00	120,000
Exhaustive	120,000	0.50	0.60	2.10	252,000

**Table 14:** Comparison of Binary-Only vs. Cascaded Multi-class Coverage Requirements.

Architecture	Conservative	Comprehensive
	Estimate	Estimate
Binary-only (detection)	4500–18,750	60,000–150,000
Cascaded (separate subtype + severity)	7650–35,625	120,000–315,000
Combined (joint subtype-severity)	6750–31,875	90,000–255,000
FDA-cleared (current)	∼700–900	[34,35]

**Table 15:** Warning Signs Suggesting Unreliable AI Performance Claims.

Warning Sign	Explanation
Perfect or near-perfect accuracy claims	Suggests data leakage, overfitting, or evaluation on trivially separable cases; real-world performance invariably lower
Single-institution validation only	Model may have learned institution-specific artifacts rather than generalizable disease patterns
No external validation	Cannot assess generalizability beyond training environment
Unreported subgroup performance	May mask significant disparities in performance across demographic groups
Validation on same data distribution as training	Does not test model’s ability to generalize to new patient populations

**Table 16:** Core Competencies for Physician–AI Collaboration.

Competency Domain	Specific Skills
AI Literacy	Understanding probabilistic vs. deterministic systems; recognizing shared limitations; knowing appropriate use cases
Critical Appraisal	Evaluating training data, validation methodology, performance metrics, failure modes, generalizability claims
Integrative Reasoning	Synthesizing AI outputs with clinical context; recognizing atypical presentations; managing competing comorbidities
Uncertainty Tolerance	Comfort with ambiguity; willingness to say “I don’t know”; appropriate use of confirmatory testing
Human Connection	Communication skills; eliciting goals of care; gathering subtle cues from patient interaction
Metacognitive Awareness	Recognizing when AI may be unreliable for the specific case; knowing when to override

**Table 17:** AI Integration Analysis Across Diagnostic Workflow Stages.

Workflow Stage	AI Contribution	Remaining Physician Role	Why AI Cannot Replace
History Taking	Ambient documentation	Eliciting subtle cues, building rapport	Requires human connection, nonverbal assessment
Differential Generation	LLM-assisted hypothesis generation	Critical evaluation, contextualization	Pattern-matching ≠ reasoning; starting point only
Physical Examination	Limited applicability	Full examination, integration with history	Tactile, dynamic assessment beyond current AI
Test Selection	Decision support algorithms	Final ordering decisions, cost-benefit analysis	Requires integration of patient-specific context
Result Interpretation	Pre-analysis, highlighting abnormalities	Final interpretation, clinical correlation	AI provides data; interpretation requires judgment
Diagnosis	Risk stratification, differential ranking	Final diagnostic determination	Population statistics ≠ individual diagnosis
Treatment Planning	Protocol suggestions, interaction checking	Final treatment decisions	Requires goals-of-care discussion, shared decision-making

**Table 18:** Classification Versus Prediction: Comparative Framework.

Dimension	Classification (Diagnosis)	Prediction (Prognosis)
Core Question	Is disease X present now?	Will disease X develop?
Temporal Orientation	Current state	Future trajectory
Output Interpretation	Binary/categorical determination	Probabilistic risk estimate
Clinical Action	Definitive treatment decision	Enhanced surveillance, prevention
Tolerance for Uncertainty	Low; requires confirmation	Higher; inherent to forecasting
Alignment with AI Nature	Strained; AI provides probabilities, but clinical use demands categorical decisions	Good; AI provides probabilities, and clinical use accepts probabilistic risk estimates
Consequence of Errors	Immediate patient harm	Missed prevention opportunity
Confirmatory Testing	Essential before treatment	Builds into monitoring protocol

**Table 19:** Comparison of classificatory versus predictive AI applications in clinical scenarios.

Clinical Domain	Classificatory Framing	Predictive Framing
Cardiology	Does this ECG show atrial fibrillation?	What is this patient’s 5-year stroke risk given AF burden?
Oncology	Does this CT show malignancy?	What is the probability of recurrence given tumor characteristics?
Radiology	Does this mammogram show cancer?	What is this patient’s 10-year breast cancer risk based on imaging features and clinical factors?
Nephrology	Does this patient have CKD Stage 3?	What is the probability of progression to ESRD within 5 years?
Psychiatry	Does this patient have major depression?	What is the probability of treatment response to SSRI vs. SNRI?

## Data Availability

All data generated or analyzed during this study are included within this article.

## Abbreviations

The following abbreviations are used in this manuscript:

AI	Artificial Intelligence
CADe	Computer-Aided Detection
CKD	Chronic Kidney Disease
CLAIM	Checklist for Artificial Intelligence in Medical Imaging
COPD	Chronic Obstructive Pulmonary Disease
CT	Computed Tomography
DL	Deep Learning
ECG	Electrocardiogram
EHR	Electronic Health Record
FDA	Food and Drug Administration
ICD-10	International Classification of Diseases, 10th Revision
LLM	Large Language Model
ML	Machine Learning
NYHA	New York Heart Association
PACS	Picture Archiving and Communication System
STARD-AI	Standards for Reporting of Diagnostic Accuracy Studies-Artificial Intelligence
TNM	Tumor, Node, Metastasis
TRIPOD+AI	Transparent Reporting of a multivariable prediction model for Individual Prognosis Or Diagnosis-Artificial Intelligence

## Appendix A. Recommendations for Stakeholders in Clinical Artificial Intelligence

To facilitate the practical translation of our theoretical findings, we present structured recommendations for clinicians, regulatory bodies, and machine learning developers. These guidelines address the operational challenges of navigating the population-to-individual gap and implementing clinical AI safely.

### Appendix A.1. Recommendations for Clinicians

1. Recognize that AI reliability on obvious cases does not imply reliability on difficult cases. When an AI system confirms what is already clinically apparent, this confirmation provides limited incremental value. The critical question is how the system performs on ambiguous presentations, atypical findings, and rare conditions. Calibrate trust accordingly.
2. Use LLMs explicitly to expand differential diagnoses. Rather than seeking AI confirmation of a leading hypothesis, use LLMs to generate comprehensive differential lists that include rare conditions you may not have considered. Treat the LLM as a tool for reducing anchoring bias, not as an independent diagnostic authority.
3. Assess patient feature fit against training data statistics. When deploying task-specific classification models for an individual patient, clinicians must evaluate whether and to what extent that specific patient’s clinical features, history, and demographics are represented within the model’s original training dataset. If the patient’s unique profile represents an out-of-distribution case, the reliability of the diagnostic prediction is entirely unknown, necessitating extreme caution.
4. Perform confirmatory testing systematically. When the differential includes conditions of varying probability, test the most likely hypothesis first but proceed to alternative hypotheses when confirmatory testing is negative or equivocal. Do not discharge patients simply because their presentation can be explained by a common condition.
5. Recognize that LLMs reproduce human biases. LLMs are trained on text written by human physicians and therefore inherit the diagnostic tendencies encoded in that text. An LLM that agrees with your initial impression may simply be reflecting the same cognitive biases that shaped your impression. Independent verification remains essential.
6. Document the diagnostic reasoning process. When AI systems contribute to clinical reasoning, document not only the AI output but the clinical reasoning that led to acceptance, modification, or rejection of that output. This documentation supports quality improvement, medicolegal defensibility, and institutional learning.

### Appendix A.2. Recommendations for Regulators

1. Require performance characterization across disease prevalence strata. Regulatory submissions should include performance metrics stratified by condition prevalence. A classifier that achieves 95% overall accuracy but fails to detect rare serious conditions may be clinically dangerous despite impressive aggregate statistics.
2. Distinguish predictive from classificatory claims. Regulatory frameworks should differentiate between AI systems marketed for predictive functions (flagging, risk stratification, hypothesis generation) and those marketed for classificatory functions (autonomous diagnosis without physician review). The latter require substantially higher evidentiary standards and may be inappropriate for conditions where rare but serious pathology must be excluded.
3. Mandate transparency regarding training data distributions. AI systems should disclose the prevalence of each diagnostic category in training data. Clinicians cannot assess the applicability of AI outputs to rare conditions without understanding how those conditions were represented during training.
4. Require validation of patient-to-model fit metrics. Regulators should mandate that clinical classification models incorporate validated auxiliary systems to evaluate patient-specific feature fit. Manufacturers must demonstrate that their software can actively quantify and report the extent to which an individual patient’s clinical features, history, and demographics align with the original training population statistics, providing explicit alerts when a patient is out of distribution.
5. Establish post-market surveillance for rare condition detection. Aggregate accuracy metrics may mask systematic failures in rare condition detection. Post-market surveillance should specifically monitor for missed diagnoses of rare but serious conditions.
6. Require labeling that communicates limitations for rare conditions. Product labeling should explicitly state that AI system performance may be reduced for conditions underrepresented in training data, and that confirmatory testing remains necessary regardless of AI output.

### Appendix A.3. Recommendations for AI Developers

1. Optimize for rare condition sensitivity, not just aggregate accuracy. Loss functions that weight all errors equally will produce systems that sacrifice rare condition detection for common condition accuracy. Consider loss functions that explicitly penalize missed rare serious conditions or that stratify performance targets by clinical significance.
2. Build active mechanisms to assess and communicate training data fit. Developers should integrate auxiliary algorithms that quantify the similarity between each new patient’s specific feature profile and the training dataset distribution. When a patient presents with clinical features, history, or demographics that place them in a poorly represented region of the model’s feature space, the system must explicitly calculate and report this distance, providing clear, clinical-facing alerts rather than generating overconfident extrapolations.
3. Design for differential diagnosis support rather than single-diagnosis output. Systems that output ranked differential diagnoses with associated probabilities support the iterative confirmatory testing workflow better than systems that output single categorical predictions. The goal should be to ensure that serious conditions appear on the differential, not to identify the single most likely diagnosis.
4. Enable verification of reasoning alignment. For image-based classifiers, attention mapping techniques should highlight the specific features driving predictions. For LLMs, outputs should include the reasoning chain that led to diagnostic suggestions. Clinicians should be able to verify that AI outputs are based on clinically relevant features rather than confounding artifacts.
5. Explicitly address training data bias toward common conditions. Training datasets should be curated or augmented to ensure adequate representation of rare serious conditions. Alternatively, systems should communicate their expected performance degradation for conditions below specified prevalence thresholds in training data.
